# Global Burden of Alcoholic Cardiomyopathy in Adults Aged 60 and Older From 1990 to 2021: A Secondary Analysis of Global Burden of Disease 2021 Data

**DOI:** 10.1002/hsr2.72358

**Published:** 2026-04-14

**Authors:** Siyi Xu, Xuqing Ying, Qian Hong, Tingru Miao, Weixun Cai, Yixin Chen, Xin Chen, Liuqin Hu

**Affiliations:** ^1^ Heart Center, Department of Electrocardiographic & Cardiac Examination, Zhejiang Provincial People's Hospital, Affiliated People's Hospital Hangzhou Medical College Hangzhou Zhejiang China

**Keywords:** alcoholic cardiomyopathy, disease burden trends, global burden of disease, the elderly

## Abstract

**Background:**

Alcoholic cardiomyopathy (ACM) poses a significant global health challenge, yet its impact on elderly populations across regions remains underexplored.

**Methods:**

Using Global Burden of Disease (GBD) 2021 data sourced from the official visualization platform, we analyzed ACM burden among individuals aged ≥ 60 years across 204 countries (1990–2021). Age‐standardized rates (ASR) for prevalence, mortality, and disability‐adjusted life years (DALYs) were calculated. Trends were assessed via estimated annual percentage change and Joinpoint regression. Future burden (2022–2050) was projected using Bayesian age‐period‐cohort models. Inequalities were measured using Slope and Concentration Indices, while frontier analysis identified optimization potential.

**Results:**

In 2021, global age‐standardized prevalence, mortality, and DALY rates were 19.33, 2.64, and 58.55 per 100,000, respectively, declining since 1990. Eastern Europe exhibited the highest burden (ASDR: 645.87), notably in Hungary, Russia, and Latvia. Males experienced 3–5 times higher rates than females. While Southern Latin America saw substantial declines, the Caribbean observed increases. A positive correlation existed between the Socio‐demographic Index (SDI) and ASRs. Decomposition analysis indicated population growth drove prevalence increases, whereas epidemiological improvements reduced mortality. Projections suggest a slight prevalence rise by 2050. Alcohol consumption remained the primary risk factor, especially in high‐SDI regions.

**Conclusion:**

Although age‐standardized ACM burden decreased among the elderly from 1990 to 2021, absolute numbers rose due to population aging and growth. Targeted interventions are needed, particularly in high‐burden regions and among males.

## 1 Introduction

Alcoholic cardiomyopathy (ACM) is an acquired form of dilated cardiomyopathy resulting from chronic excessive alcohol consumption [1]. It is characterised primarily by ventricular dilation, impaired contractile function, and thinning of the ventricular walls [2]. As a significant subtype of non‐ischemic cardiomyopathy, ACM accounts for approximately 10% of all dilated cardiomyopathy cases [3]. The underlying pathophysiological mechanisms involve direct cardiotoxic effects of ethanol and its metabolites, leading to disruption of membrane integrity [4], ion channel dysfunction [5], suppression of protein synthesis [6], and apoptosis via the BAX/BCL‐2 pathway [7].

Globally, alcohol consumption continues to rise, posing a substantial public health challenge [8] [9]. Importantly, ACM development is dose‐ and duration‐dependent, with no established safe threshold for alcohol intake, underscoring the importance of population‐wide alcohol control strategies [10].

The assessment of ACM burden remains a complex undertaking from a methodological perspective [11]. Conventional methods that depend on alcohol‐attributable fractions (AAFs) are constrained by the absence of a reliable exposure‐disease relationship [11]. The Global Burden of Disease (GBD) study has enhanced mortality estimates through systematic redistribution of garbage‐coded deaths [12]. Yet, significant gaps persist in understanding the burden among older adults of ACM.

It is important to note that elderly individuals are more vulnerable to ACM due to age‐related physiological changes, including reduced ethanol metabolism, increased cardiac susceptibility, and a higher prevalence of comorbidities such as hypertension and diabetes [1, 13]. The combination of these factors with patterns of prolonged and often heavy alcohol use in some elderly populations may accelerate disease progression and worsen outcomes. Moreover, the demographic ageing that is occurring on a global scale serves to heighten the urgency of addressing alcohol‐related heart disease in this particular age group [14, 15].

Whilst earlier research has utilised GBD data to examine ACM burden, the majority of these studies have concentrated on general adult populations or specific geographic areas [16]. This has resulted in a significant absence of detailed, age‐specific analyses among the elderly [17, 18]. Utilising data from GBD 2021, the present study aims to comprehensively evaluate the global, regional, and national burden of ACM among adults aged 60 and older from 1990 to 2021 [12]. We employ a range of advanced analytical approaches, including trend analysis, health inequality metrics, decomposition, and predictive modelling, to elucidate the impact of socioeconomic, demographic, and cultural factors on the burden of ACM in the elderly. The aim is to inform targeted prevention and clinical strategies.

## 2 Methods

### 2.1 GBD 2021 Overview

The GBD 2021 study employed refined standardization methodologies to generate updated epidemiological estimates for 371 diseases and injuries, as well as 88 risk factors, across 204 countries and territories and 21 GBD regions from 1990 to 2021. As a secondary analysis of de‐identified, publicly available data, this study did not require ethics committee approval or informed consent. All raw data within the GBD framework were systematically mapped to standard International Classification of Diseases (ICD) codes. Furthermore, non‐specific or ill‐defined codes, commonly referred to as “garbage codes,” underwent systematic redistribution. Data quality was rigorously evaluated and adjusted in accordance with pre‐established standardized protocols. Missing data were imputed using a consistent modeling strategy, and all rate metrics were age‐standardized to remove bias arising from variations in population age structures and to ensure comparability.

### 2.2 Data Sources and Extraction

This study utilized data from the Global Burden of Disease 2021 (GBD 2021) study [12]. Data were extracted via the GBD visualization tool (https://vizhub.healthdata.org/gbd-results/) and analyzed by geography, demographic subgroups, and core burden metrics. Geographic stratification included global, Socio‐Demographic Index (SDI)‐based (five categories), and 21 GBD regions [12, 19]. The analysis focused on individuals aged 60 years and older, categorized into eight 5‐year age groups:60–64, 65–69, 70–74, 75–79, 80–84, 85–89, 90–94, and 95 + , with subgroup analysis by sex (male, female, and total). Key outcomes—prevalence, mortality, and disability‐adjusted life years (DALYs) for alcoholic cardiomyopathy (ACM)—were extracted with 95% uncertainty intervals. ACM cases were identified using the ICD‐10 code I42.6 to ensure consistency and comparability. Mortality attribution adhered to GBD standards, distinguishing between ACM as an underlying cause versus deaths related to complications through ICD mapping, underlying cause‐of‐death principles, and statistical algorithms. All data processing and analysis were conducted using R software (version 4.4.2).

### 2.3 Statistical Analysis Framework

#### 2.3.1 Descriptive Statistics of Disease Burden

To eliminate the impact of population age structure differences, the prevalence, mortality, and DALYs for the population aged 60 and older were age‐standardized using the GBD 2021 global standard population structure. The age‐standardized prevalence rate (ASPR), age‐standardized mortality rate (ASMR), and age‐standardized DALYs rate (ASDR) were calculated along with their corresponding 95% UI. Spearman's rank correlation coefficient was then used to assess the strength of association between the SDI and these age‐standardized rates (ASR).

#### 2.3.2 Temporal Trend Analysis

This study used both the Estimated Annual Percentage Change (EAPC) and Joinpoint regression analysis to assess the temporal trends in disease burden [20, 21]. EAPC was calculated based on a log‐linear regression model (ln(Y) = α + βX + ε), with the formula 100× [exp(β)−1], where β represents the regression coefficient of the year variable X and Y denotes the natural log of ASR. EAPC values and their 95% confidence intervals (CI) were used to determine the significance and direction of trends (EAPC > 0 with the 95% CI lower limit > 0 indicates an increasing trend, EAPC < 0 with the 95% CI upper limit < 0 indicates a decreasing trend). Joinpoint regression analysis was implemented using the Joinpoint Regression Program 4.9.1.0 software, with Monte Carlo permutation tests used to identify significant inflection points in trends and calculate the annual average percentage change (AAPC) over the study period.

#### 2.3.3 Decomposition Analysis of Disease Burden

To quantify the relative contributions of population aging, population size growth, and epidemiological changes in alcoholic cardiomyopathy to the total burden variation over the observation period, this study performed a demographic decomposition analysis, following the methodology outlined in the established literature framework [22, 23].

#### 2.3.4 Bayesian Age‐Period‐Cohort (BAPC) Analysis

For future trend prediction, a Bayesian Age‐Period‐Cohort (BAPC) model was constructed to forecast the disease burden from 2022 to 2050. The model applied a second‐order random walk before smoothing the age, period, and cohort effects to avoid overfitting, and the Integrated Nested Laplace Approximation (INLA) method was used to efficiently compute the posterior marginal distributions of the model parameters, significantly improving computational efficiency. The robustness of the model predictions was validated through cross‐validation [24].

#### 2.3.5 Health Inequality Analysis and Frontier Analysis

Health inequality was quantified using the Slope Index of Inequality (SII) and Concentration Index (CII) [25]. The SII measures the absolute differences in median ASDR values across population groups ordered by SDI, while the CII, based on the Lorenz curve, reflects the relative inequality in ASDR burden with respect to SDI distribution. Additionally, frontier analysis based on SDI was conducted to construct a “frontier curve” reflecting the theoretically achievable minimum ASDR at each specific SDI level. This analysis used local weighted regression (LOESS) combined with polynomial fitting to plot boundary curves and adjusted the smoothing parameter to capture the nonlinear relationship between ASDR and SDI. To ensure robustness, 100 bootstrap resampling iterations were performed to calculate the average ASDR at each SDI node. By comparing the actual ASDR of each country/region with the theoretical frontier value at its corresponding SDI level, potential areas for improvement in controlling the burden of alcoholic cardiomyopathy were identified [25].

#### 2.3.6 Statistical Reporting and Guidelines

The design, analysis, and reporting of this study adhere to established international guidelines to ensure transparency, reproducibility, and methodological rigor. We followed the recommendations of the CONSORT 2025 Statement [26] for structured reporting, the SAMPL guidelines [27] for basic statistical reporting in biomedical journals, and the Guidelines for reporting of statistics for clinical research in urology [28] for proper analysis, reporting, and interpretation of clinical research data.

All statistical terms, abbreviations, and symbols used in this manuscript are clearly defined upon first appearance in the text, tables, and figures. The analyses were pre‐specified based on the GBD study framework, including descriptive epidemiology (age‐standardized rates with 95% uncertainty intervals), temporal trend analysis (estimated annual percentage change and Joinpoint regression), and health inequality assessment (Slope Index of Inequality and Concentration Index). Exploratory analyses, including decomposition analysis, frontier analysis, and Bayesian age‐period‐cohort projections, are explicitly identified as such and are intended to generate hypotheses for future research.

## 3 Results

### 3.1 Global Burden

**Figure 1:**
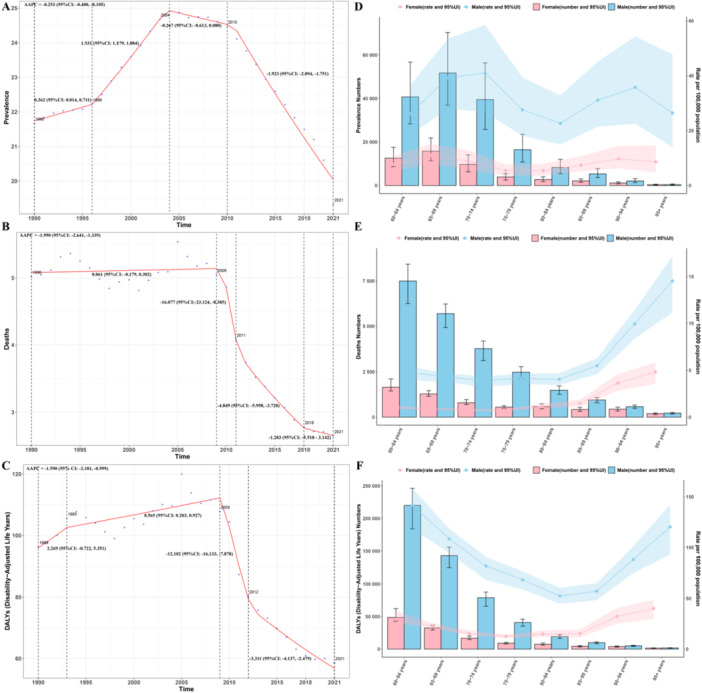
Trends in age‐standardized rates of alcoholic cardiomyopathy among adults aged ≥ 60 years, 1990–2021. (A–C) Joinpoint regression analysis of age‐standardized prevalence rate (ASPR; A), mortality rate (ASMR; B), and disability‐adjusted life years rate (ASDR; C) from 1990 to 2021. Solid lines represent fitted trends; symbols (•) indicate observed data points. Vertical dashed lines mark the years where trends changed significantly (inflection points) as identified. (D–F) Age‐ and sex‐specific trends in ASPR (D), ASM (E), and ASDR (F) across eight 5‐year age groups in 2021. Blue lines represent males; red lines represent females. Shaded areas indicate 95% uncertainty intervals. Asdr, age‐standardized disability‐adjusted life years rate; Asmr, age‐standardized mortality rate; Aspr, age‐standardized prevalence rate; UI, uncertainty interval.

In 2021, the age‐standardized prevalence rate (ASPR) of alcoholic cardiomyopathy among individuals aged 60 and older globally was 19.33 (95% UI: 13.23–27.11) per 100,000, representing a 10.80% decrease from 1990. The age‐standardized mortality rate (ASMR) was 2.64 (95% UI: 2.27–2.94) per 100,000, reflecting a 47.41% decrease, and the age‐standardized DALYs rate (ASDR) was 58.55 (95% UI: 50.73–64.65) per 100,000 years, showing a reduction of 39.16% (Table 1 and Supporting Information S1: Figure 1A–C). In 2021, the total number of elderly individuals with alcoholic cardiomyopathy was 213,068.94 (95% UI: 146,023.08–298,548.99), with 28,623.88 (95% UI: 24,628.27–31,763.66) deaths, and the total DALYs were 643,857.06 (95% UI: 558,152.92–710,379.19) years. EAPC analysis showed that from 1990 to 2021, the ASPR decreased, but this trend was not statistically significant, while both ASMR and ASDR decreased significantly (Table 1 and Supporting Information S1: Figure 1E‐F). Further AAPC analysis indicated a significant decline in all three ASRs. Specifically, ASPR showed inflection points in 1996, 2004, and 2010, with a continuous decrease from 2010 onwards; ASMR showed inflection points in 2009, 2011, and 2018, with a significant decline from 2018 onwards; ASDR showed inflection points in 1993, 2009, and 2012, followed by a sustained decline from 2012 (Figure 1A–C).

**Table 1:** Age‐standardized prevalence, mortality, and DALY rates of alcoholic cardiomyopathy with corresponding EAPC trends among adults aged ≥ 60 years, by region, 1990–2021.

location	ASPR			ASMR			ASDR		
	1990 (per 100,000 population, 95% UI)	2021 (per 100,000 population, 95% UI)	EAPCs (95% CI)	1990 (per 100,000 population, 95% UI)	2021 (per 100,000 population, 95% UI)	EAPCs (95% CI)	1990 (per 100,000 population, 95% UI)	2021 (per 100,000 population, 95% UI)	EAPCs (95% CI)
Global	21.67 (14.54, 30.50)	19.33 (13.23, 27.11)	−0.44 (−1.49, 0.62)	5.02 (4.59, 5.48)	2.64 (2.27, 2.94)	−2.61 (−3.69, −1.52)	96.24 (89.36, 104.36)	58.55 (50.73, 64.65)	−2.22 (−3.37, −1.06)
High SDI	37.93 (25.61, 53.06)	42.47 (29.59, 58.99)	0.11 (−1.41, 1.65)	7.06 (6.21, 7.92)	3.46 (3.11, 3.78)	−3.38 (−5.12, −1.61)	140.09 (124.61, 156.17)	77.71 (70.67, 84.21)	−2.94 (−4.71, −1.13)
High‐middle SDI	33.51 (22.34, 48.11)	30.09 (19.81, 43.32)	−0.30 (−0.79, 0.19)	9.50 (8.64, 10.35)	6.40 (5.47, 7.25)	−1.76 (−2.71, −0.81)	182.22 (167.94, 196.93)	146.79 (126.31, 165.18)	−1.34 (−2.36, −0.30)
Middle SDI	3.14 (2.16, 4.42)	3.92 (2.62, 5.62)	0.81 (−0.86, 2.51)	0.74 (0.55, 1.02)	0.54 (0.31, 0.72)	−1.42 (−2.87, 0.05)	15.44 (11.73, 20.88)	11.00 (6.59, 14.64)	−1.55 (−3.19, 0.13)
Low‐middle SDI	3.36 (2.30, 4.75)	2.85 (1.87, 4.11)	−0.57 (−2.25, 1.14)	0.75 (0.42, 1.23)	0.54 (0.26, 0.96)	−1.27 (−2.70, 0.18)	15.55 (8.94, 25.18)	11.13 (5.56, 19.59)	−1.29 (−2.92, 0.37)
Low SDI	3.76 (2.22, 5.93)	3.83 (2.24, 6.06)	0.50 (−1.56, 2.60)	0.43 (0.09, 0.93)	0.36 (0.07, 0.83)	−0.48 (−1.44, 0.49)	8.45 (2.27, 17.86)	7.34 (1.89, 16.61)	−0.34 (−1.65, 0.99)
Andean Latin America	0.31 (0.21, 0.44)	0.33 (0.22, 0.48)	1.01 (0.49, 1.54)	0.09 (0.03, 0.17)	0.04 (0.01, 0.07)	−2.13 (−2.70, −1.54)	1.50 (0.54, 2.84)	0.65 (0.19, 1.21)	−1.97 (−2.55, −1.39)
Australasia	40.68 (28.47, 56.64)	98.21 (68.60, 133.83)	2.29 (0.52, 4.10)	5.95 (4.87, 7.18)	4.11 (3.33, 4.94)	−1.71 (−3.57, 0.18)	120.19 (100.35, 142.54)	92.01 (75.86, 109.74)	−1.36 (−3.36, 0.68)
Caribbean	11.61 (8.18, 15.93)	59.21 (40.75, 83.58)	6.61 (3.71, 9.58)	2.12 (1.42, 3.45)	8.18 (6.38, 10.26)	5.46 (2.98, 8.00)	48.34 (32.38, 78.21)	177.54 (139.23, 221.60)	5.26 (2.87, 7.70)
Central Asia	7.61 (4.86, 10.96)	9.73 (6.12, 14.27)	0.74 (−0.15, 1.63)	2.66 (2.12, 3.35)	3.07 (2.47, 3.87)	0.04 (−1.18, 1.27)	53.46 (43.25, 66.45)	65.43 (52.50, 82.91)	0.21 (−1.06, 1.48)
Central Europe	45.78 (30.27, 65.35)	68.39 (47.43, 95.21)	1.47 (0.06, 2.91)	14.35 (12.49, 16.64)	11.68 (9.26, 13.75)	−1.19 (−2.73, 0.37)	252.19 (220.80, 289.19)	249.00 (198.26, 291.15)	−0.56 (−2.15, 1.06)
Central Latin America	5.26 (3.67, 7.33)	5.25 (3.50, 7.55)	−0.32 (−2.08, 1.47)	0.98 (0.81, 1.17)	0.63 (0.54, 0.74)	−2.15 (−4.05, −0.20)	21.13 (17.55, 25.19)	13.72 (11.73, 16.00)	−2.13 (−4.16, −0.06)
Central Sub‐Saharan Africa	4.09 (2.05, 7.04)	3.56 (1.82, 6.12)	0.22 (−2.01, 2.51)	0.02 (0.00, 0.09)	0.01 (0.00, 0.07)	−0.68 (−2.05, 0.70)	0.70 (0.28, 2.10)	0.58 (0.24, 1.78)	−0.26 (−2.08, 1.60)
East Asia	1.52 (1.02, 2.18)	2.60 (1.61, 3.96)	2.11 (0.92,3.32)	0.25 (0.11, 0.51)	0.32 (0.06, 0.54)	0.87 (−0.13, 1.88)	4.63 (2.15, 9.62)	6.20 (1.39, 10.00)	0.99 (−0.19, 2.17)
Eastern Europe	94.13 (62.62, 135.96)	128.27 (83.69, 186.25)	1.30 (0.68, 1.92)	24.77 (22.58, 27.07)	27.62 (23.74, 31.57)	−0.02 (−1.23, 1.21)	493.05 (455.29, 533.98)	645.87 (559.47, 731.83)	0.41 (−0.88, 1.72)
Eastern Sub‐Saharan Africa	4.97 (2.62, 8.34)	4.58 (2.43, 7.66)	0.03 (−3.68,3.88)	0.00 (0.00, 0.03)	0.00 (0.00, 0.02)	−0.95 (−2.75, 0.89)	0.55 (0.26, 1.11)	0.49 (0.23, 1.01)	−0.27 (−3.45, 3.02)
High‐income Asia Pacific	7.76 (4.30, 12.20)	7.69 (4.67, 11.57)	−0.48 (−2.65,1.73)	1.23 (1.07, 1.41)	0.55 (0.48, 0.63)	−3.48 (−5.91, −0.98)	26.57 (23.30, 30.52)	12.11 (10.59, 13.72)	−3.40 (−5.97, −0.77)
High‐income North America	45.15 (29.00, 65.36)	51.97 (35.34, 73.27)	−0.11 (−1.89,1.70)	6.65 (5.88, 7.54)	4.43 (4.00,4.83)	−2.15 (−4.23, −0.02)	143.88 (127.35, 163.44)	101.19 (92.61, 110.04)	−1.95 (−4.01, 0.14)
North Africa and Middle East	0.94 (0.66, 1.30)	0.86 (0.58, 1.24)	−0.14 (−0.92, 0.64)	0.32 (0.06, 0.77)	0.21 (0.05, 0.49)	−1.25 (−1.41, −1.10)	5.85 (1.27, 13.70)	3.69 (0.94, 8.72)	−1.43 (−1.89, −0.97)
Oceania	0.87 (0.61, 1.21)	0.76 (0.54,1.06)	−0.71 (−1.35, −0.07)	0.32 (0.04, 0.72)	0.22 (0.03, 0.56)	−1.57 (−2.03, −1.11)	6.65 (0.94, 14.98)	4.47 (0.72, 11.74)	−1.64 (−2.25, −1.03)
South Asia	1.73 (1.18, 2.47)	1.74 (1.10,2.57)	0.21 (−1.57, 2.02)	0.44 (0.09, 1.03)	0.39 (0.07, 0.91)	−0.17 (−1.74, 1.41)	9.18 (1.96, 21.29)	7.94 (1.51, 18.19)	−0.35 (−2.03, 1.35)
Southeast Asia	0.91 (0.64, 1.27)	0.97 (0.66, 1.37)	−0.24 (−1.89, 1.45)	0.23 (0.04, 0.50)	0.19 (0.03, 0.36)	−0.97 (−2.31, 0.39)	4.54 (1.04, 10.29)	3.76 (0.76, 7.22)	−1.13 (−2.73, 0.51)
Southern Latin America	18.45 (11.64, 26.85)	6.98 (4.27, 10.33)	−3.81 (−6.48, −1.07)	5.61 (4.22, 7.35)	1.01 (0.81, 1.24)	−6.18 (−9.03, −3.24)	122.33 (92.15, 159.49)	21.55 (17.54, 26.21)	−6.29 (−9.18, −3.30)
Southern Sub‐Saharan Africa	0.66 (0.36, 1.11)	0.50 (0.27, 0.83)	−0.51 (−1.68, 0.68)	0.08 (0.01, 0.16)	0.05 (0.01, 0.10)	−0.81 (−1.26, −0.37)	0.79 (0.18, 1.63)	0.56 (0.12, 1.11)	−0.79 (−1.32, −0.25)
Tropical Latin America	23.10 (15.48, 33.00)	12.90 (8.20, 19.36)	−2.39 (−4.53, −0.20)	5.54 (4.73, 6.52)	1.33 (1.17, 1.49)	−5.59 (−7.83, −3.30)	127.07 (109.02, 149.21)	31.35 (27.76, 34.94)	−5.57 (−7.95, −3.12)
Western Europe	35.61 (23.90, 49.39)	40.91 (27.85, 58.03)	0.44 (−0.96, 1.86)	7.70 (6.44, 8.98)	3.03 (2.55, 3.51)	−4.26 (−5.88, −2.61)	146.94 (124.18, 169.90)	66.18 (56.58, 75.62)	−3.80 (−5.46, −2.10)
Western Sub‐Saharan Africa	4.92 (2.61, 8.24)	5.28 (2.82, 8.84)	0.75 (−0.75, 2.28)	0.51 (0.03, 1.03)	0.21 (0.02, 0.47)	−3.28 (−3.48, −3.09)	8.19 (0.95, 17.00)	3.79 (0.67, 9.24)	−2.96 (−3.92, −1.99)

Abbreviations: ASDR, age‐standardized disability‐adjusted life years rate (per 100,000 population); ASMR, age‐standardized mortality rate (per 100,000 population); ASPR, age‐standardized prevalence rate (per 100,000 population); CI, confidence interval; DALYs, disability‐adjusted life years; EAPC, estimated annual percentage change; GBD, Global Burden of Disease; SDI, Socio‐demographic Index; UI, uncertainty interval.Notes: interval. Notes: Data are presented with 95% uncertainty intervals (UI) in parentheses. EAPC values with 95% confidence intervals (CI) indicate the average annual change over the 1990–2021 period. EAPC > 0 with 95% CI lower limit > 0 indicates a statistically significant increasing trend; EAPC < 0 with 95% CI upper limit < 0 indicates a statistically significant decreasing trend.

### 3.2 Regional Burden

Among the five SDI regions, in 2021, the High SDI region had the highest ASPR (42.47, 95% UI: 29.59–58.99 per 100,000), while the High‐middle SDI region had the highest ASMR (6.40, 95% UI: 5.47–7.25 per 100,000) and ASDR (146.79, 95% UI: 126.31–165.18 per 100,000). In contrast, the lowest ASPR was found in the Low‐middle SDI region (2.85, 95% UI: 1.87–4.11 per 100,000), the lowest ASMR in the Low SDI region (0.36, 95% UI: 0.07–0.83 per 100,000), and the lowest ASDR in the Low SDI region (7.34, 95% UI: 1.89–16.61 per 100,000) (Table 1 and Supporting Information S1: Figure 1A–C). From 1990 to 2021, only the High SDI and High‐middle SDI regions showed a significant decrease in ASMR and ASDR, while other SDI regions exhibited no significant changes (Table 1 and Supporting Information S1: Figure 1D–F).

Among the 21 GBD regions, Eastern Europe ranked the highest in all three indicators: ASPR was 128.27 (95% UI: 83.69–186.25), ASMR was 27.62 (95% UI: 23.74–31.57), and ASDR was 645.87 (95% UI: 559.47–731.83) per 100,000. In contrast, the lowest ASPR was found in Andean Latin America (0.33, 95% UI: 0.22–0.48), the lowest ASMR in Central Sub‐Saharan Africa (0.01, 95% UI: 0.00–0.07), and the lowest ASDR in Eastern Sub‐Saharan Africa (0.49, 95% UI: 0.23–1.01) (Table 1 and Supporting Information S1: Figure 1A–C). From 1990 to 2021, the Caribbean was the only region where all three ASRs showed a significant increase (EAPC of 6.61%, 5.46%, and 5.26%, respectively). In contrast, South Latin America saw significant declines in all three ASRs (EAPC of −3.81%, −6.18%, and −6.29%), with the annual reduction in ASMR and ASDR exceeding 5.5% in Tropical Latin America. In high‐income regions, Western Europe saw a slight increase in ASPR (0.44%) but a sharp decline in ASMR (−4.26%), while the High‐income Asia‐Pacific region experienced reductions of approximately −3.5% per year in both ASMR and ASDR. Furthermore, significant reductions in ASMR and ASDR were observed in Oceania, North Africa and the Middle East, and West Africa, while no notable changes were observed in East Africa, Central Africa, South Asia, and Southeast Asia (Table 1 and Supporting Information S1: Figure 1D–F).

### 3.3 National Burden

**Figure 2:**
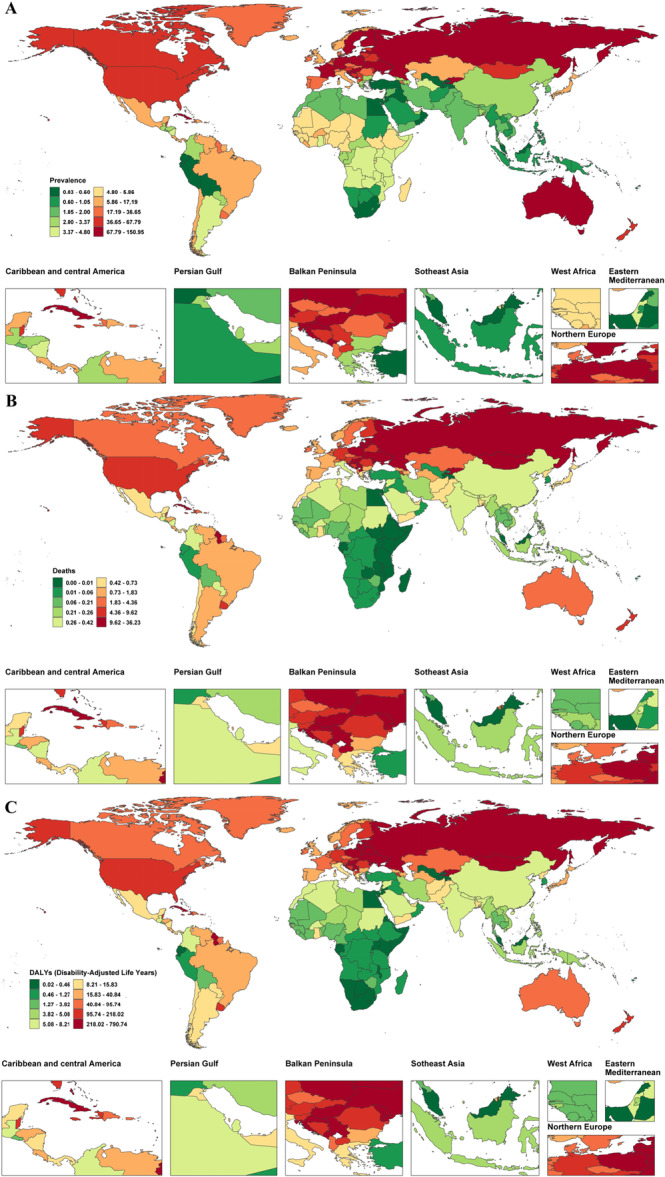
Global distribution of age‐standardized prevalence, mortality, and DALY rates of alcoholic cardiomyopathy among adults aged ≥ 60 years, 2021. (A) Age‐standardized prevalence rate (ASPR) of alcoholic cardiomyopathy per 100,000 population aged ≥ 60 years in 2021. Darker colors indicate higher burden. (B) Age‐standardized mortality rate (ASMR) per 100,000 population aged ≥ 60 years in 2021. (C) Age‐standardized DALY rate (ASDR) per 100,000 population aged ≥ 60 years in 2021. ASDR, age‐standardized disability‐adjusted life years rate (per 100,000 population); ASMR, age‐standardized mortality rate (per 100,000 population); ASPR, age‐standardized prevalence rate (per 100,000 population); DALYs, disability‐adjusted life years.

**Figure 3:**
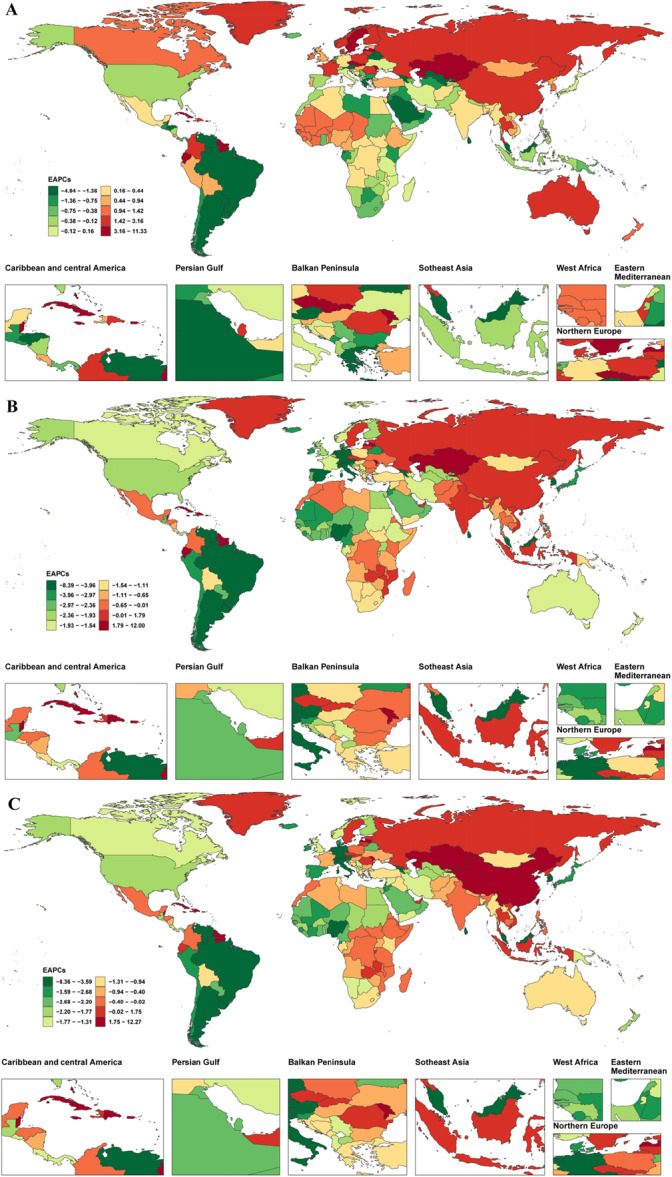
Estimated annual percentage changes in age‐standardized prevalence, mortality, and DALY rates of alcoholic cardiomyopathy among adults aged ≥ 60 years, 1990–2021. (A) Estimated annual percentage change (EAPC) in age‐standardized prevalence rate (ASPR) from 1990 to 2021. Red shades indicate increasing trends; green shades indicate decreasing trends. (B) EAPC in age‐standardized mortality rate (ASMR) from 1990 to 2021. (C) EAPC in age‐standardized DALY rate (ASDR) from 1990 to 2021. ASDR, age‐standardized disability‐adjusted life years rate; ASMR, age‐standardized mortality rate; ASPR, age‐standardized prevalence rate; CI, confidence interval; EAPC, estimated annual percentage change.

At the national level, in 2021, Hungary (ASPR 126.23 per 100,000; ASMR 35.87 per 100,000; ASDR 782.91 per 100,000), Russia (ASPR 149.46 per 100,000; ASMR 29.36 per 100,000; ASDR 716.63 per 100,000), and Latvia (ASPR 130.97 per 100,000; ASMR 29.74 per 100,000; ASDR 725.83 per 100,000) ranked among the top three for all indicators, with ASDR values more than 12 times the global average. In contrast, the countries with the lowest ASPR included Malaysia (0.03 per 100,000), Egypt (0.07 per 100,000), and Uzbekistan (0.07 per 100,000); the countries with the lowest ASDR were Tajikistan (0.05 per 100,000), Egypt (0.08 per 100,000), and Uzbekistan (0.31 per 100,000). Notably, 21 countries (e.g., Burundi, Rwanda) had an ASMR close to zero (95% UI upper limit ≤ 0.03), while countries such as Bosnia and Herzegovina showed wide confidence intervals for ASDR (UI: 34.62–423.80), suggesting data uncertainty (Figure 2A–C and Supporting Information S2: Table 1). From 1990 to 2021, the countries with the fastest increase in ASPR were Kazakhstan (11.22%), Bermuda (9.44%), and Saint Kitts and Nevis (7.54%), while the countries with the greatest decrease were Mauritius (−4.89%), Sri Lanka (−3.13%), and Georgia (−4.42%). The countries with the fastest increase in ASMR were Kazakhstan (11.88%), Cuba (10.25%), and Moldova (3.81%), and the fastest decrease was observed in Singapore (−6.96%), Mauritius (−8.31%), and the Netherlands (−5.46%). The countries with the fastest increase in ASDR were Kazakhstan (12.15%), Cuba (10.15%), and Guyana (5.40%), while the fastest decrease was observed in Mauritius (−8.28%), Singapore (−7.04%), and Sri Lanka (−5.29%). Notably, Kazakhstan ranked first in all three ASRs, while Mauritius ranked first in the largest decline in ASPR and ASDR (Figure 3A–C and Supporting Information S2: Table 1).

### 3.4 Age‐Sex‐Time Trends

Age‐sex trend analysis indicated that male ASPR was more than three times higher than female ASPR across all age groups, with the peak occurring at 70‐74 years (41.00 per 100,000 vs. female peak 10.98 per 100,000 at 65–69 years). The gender disparity in ASMR widened with age, with males aged 60–64 (4.82 per 100,000) being 4.8 times higher than females (1.00 per 100,000), and the difference remained at three times higher at 95+ years (14.54 per 100,000 vs. 4.84 per 100,000). ASDR significantly increased after age 80, with the male 95+ age group showing a twofold increase in ASDR (120.23 per 100,000) compared to the 85–89 age group (57.22 per 100,000), while the increase in females was 161.5%. Throughout all age groups, male ASDR was 3–5 times higher than female ASDR, highlighting the high‐risk profile of elderly males (Figure 1D–F). Age‐time trend analysis revealed that male ASPR increased from 30.58 per 100,000 in 1990 to a peak of 39.61 per 100,000 in 2005, before decreasing to 32.26 per 100,000 in 2021; female ASPR showed a continuous decline from 14.58 per 100,000 in 1990 to 8.22 per 100,000 in 2021. Male ASMR peaked at 8.52 per 100,000 in 2005 and decreased to 4.58 per 100,000 by 2021, while female ASMR steadily declined from 3.28 per 100,000 in 1990 to 1.01 per 100,000 in 2021. Similarly, male ASDR remained higher, with female ASDR decreasing significantly from 57.94 per 100,000 in 1990 to 21.33 per 100,000 in 2021 (Supporting Information S1: Figure 2A–C). Age‐time trend analysis showed that the ASR for all age groups decreased over time, with the 95+ age group consistently having the highest ASMR, and its ASDR being higher than other age groups before 2000. After 2000, the 60–64 age group had a higher ASMR than the 95+ age group. ASPR values were relatively similar across age groups, with fluctuations over time (Supporting Information S1: Figure 2D–F).

### 3.5 Correlation Between ASR and SDI

**Figure 4:**
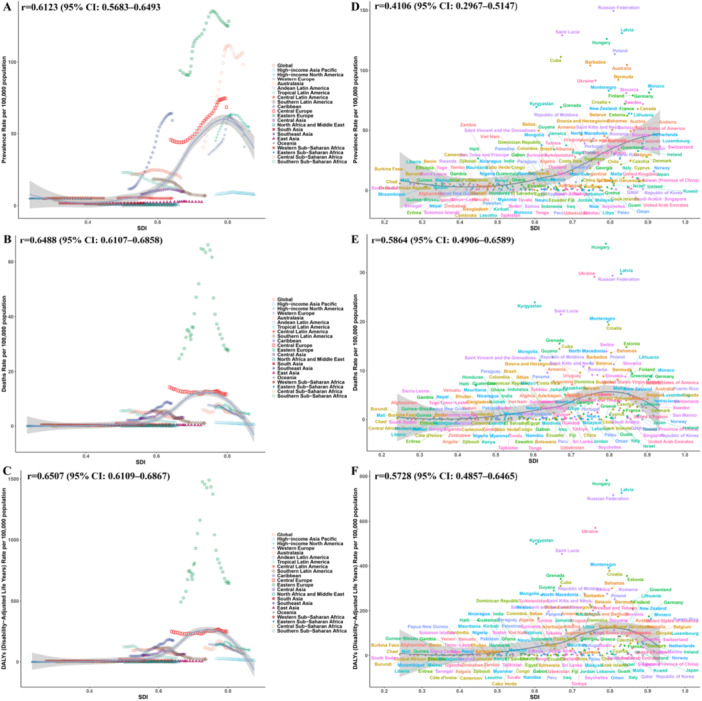
Correlation between age‐standardized rates of alcoholic cardiomyopathy and Socio‐demographic Index among adults aged ≥ 60 years, 2021. (A–C) Correlation between age‐standardized prevalence rate (ASPR; A), mortality rate (ASMR; B), and DALY rate (ASDR; C) with Socio‐demographic Index (SDI) across 21 GBD regions in 2021. Each point represents a region, with point size proportional to population size. The blue line represents the fitted non‐linear relationship (LOESS regression), with shaded areas indicating 95% confidence bands. (D–F) Correlation between ASPR (D), ASMR (E), and ASDR (F) with SDI across 204 countries and territories in 2021. Each point represents a country. ASDR, age‐standardized disability‐adjusted life years rate (per 100,000 population); ASMR, age‐standardized mortality rate (per 100,000 population); ASPR, age‐standardized prevalence rate (per 100,000 population); DALYs, disability‐adjusted life years; GBD, Global Burden of Disease; LOESS, locally estimated scatterplot smoothing; SDI, Socio‐demographic Index.

There was a significant positivecorrelation between the ASR of elderly alcoholic cardiomyopathy and SDI. Specifically, the ASR gradually increased with the increase in SDI, with acceleration beginning when SDI approached 0.6, and a decline in ASR when SDI neared 0.8. This trend was consistent across 21 regions (Figure 4A–C) and 204 countries (Figure 4D–F).

**Figure 5:**
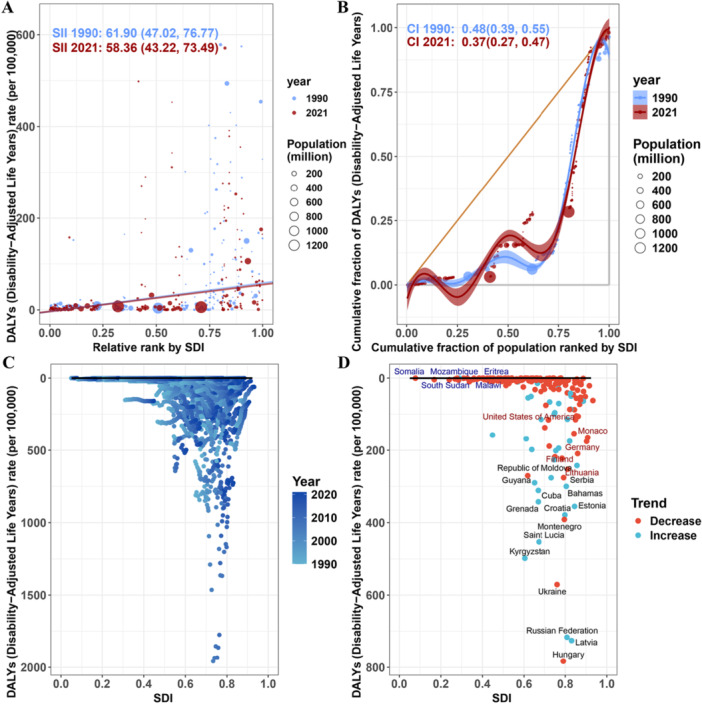
Health inequality trends and frontier analysis of alcoholic cardiomyopathy burden among adults aged ≥ 60 years, 1990–2021. (A) Slope Index of Inequality (SII) for ASDR across SDI levels, 1990 and 2021. (B) Concentration Index (CII) for ASDR relative to SDI, 1990–2021. (C) Temporal trends in age‐standardized DALY rates (ASDR) for 204 countries, 1990–2021. (D) Frontier analysis gap between observed and optimal ASDR by SDI level, 2021. ASDR, age‐standardized disability‐adjusted life years rate (per 100,000 population); CI, confidence interval; CII, Concentration Index; DALYs, disability‐adjusted life years; LOESS, locally estimated scatterplot smoothing; SDI, Socio‐demographic Index; SII, Slope Index of Inequality.

Health inequality analysis showed significant absolute and relative inequalities in ASDR across SDI levels. The Slope Index indicated that from 1990 to 2021, the ASDR gap between the highest and lowest SDI countries narrowed from 61.90 (95% UI: 47.02–76.77) per 100,000 years to 58.36 (95% UI: 43.22–73.49) per 100,000 years (Figure 5A), suggesting a reduction in absolute inequality, with disease burden concentrated in high‐SDI countries. In terms of relative inequality, the Concentration Index narrowed from 0.48 (95% CI: 0.39–0.55) in 1990 to 0.37 (95% CI: 0.27–0.47) in 2021 (Figure 5B), indicating that while the disparity between high‐SDI and low‐SDI countries has diminished, the disease burden remains predominantly in high‐SDI countries.

Frontier analysis showed that most countries experienced a decline in ASDR from 1990 to 2021 (Figure 5C). The 15 countries with the greatest discrepancy from the optimal disease burden included Hungary, Latvia, the Russian Federation, Ukraine, Kyrgyzstan, Saint Lucia, Montenegro, Croatia, Grenada, Estonia, Cuba, Serbia, the Bahamas, Guyana, and Mongolia (range of potential improvement: 12.14–35.87). In low‐SDI countries (SDI < 0.50), countries with the smallest disparity from the frontier included Ethiopia, Uganda, Somalia, Comoros, and Rwanda, while in high‐SDI countries (SDI > 0.85), the countries with the largest frontier differences were Lithuania, Finland, Monaco, Germany, and Austria (Figure 5D).

### 3.6 Decomposition Analysis Results

**Figure 6:**
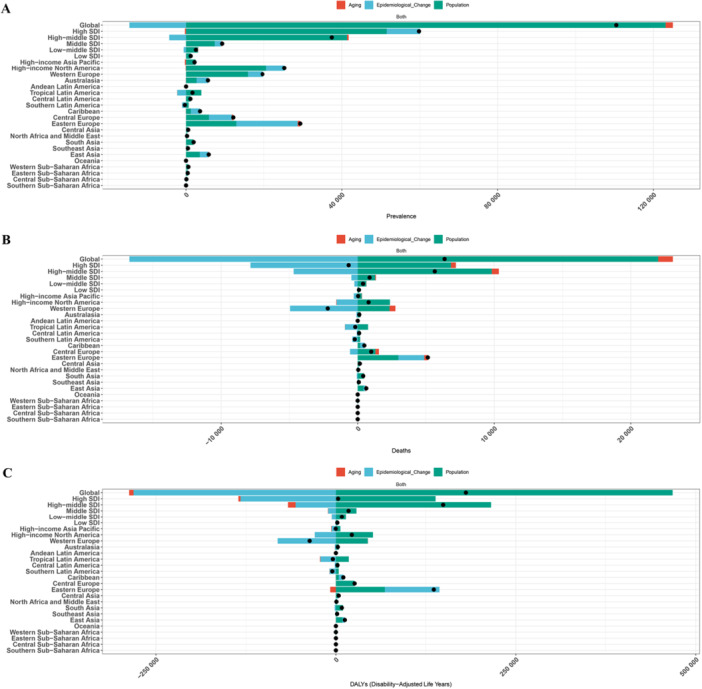
Decomposition analysis of changes in alcoholic cardiomyopathy burden among adults aged ≥ 60 years, by region and SDI level, 1990–2021. (A) Decomposition of changes in prevalence (number of cases) from 1990 to 2021. Bars represent the absolute contribution of three components: population aging (orange), population growth (blue), and epidemiological changes (green). Positive values indicate factors contributing to an increase in burden; negative values indicate factors contributing to a decrease. (B) Decomposition of changes in mortality (number of deaths) from 1990 to 2021.C: Decomposition of changes in disability‐adjusted life years (DALYs) from 1990 to 2021. ASDR, age‐standardized disability‐adjusted life years rate; ASMR, age‐standardized mortality rate; ASPR, age‐standardized prevalence rate; DALYs, disability‐adjusted life years; SDI, Socio‐demographic Index.

Decomposition analysis showed that globally, the increase in prevalence was primarily driven by population growth, with a contribution of 111.43%. Aging contributed 1.68%, while epidemiological changes had a negative impact (−13.11%). In Eastern Europe, the aging effect was most prominent, contributing 53.81%, while population growth drove the increase in Western Europe (80.94%) and high‐income North America (81.51%). Changes in mortality were more complex, with global mortality growth of 345.21% mainly driven by population growth, aging contributing 16.81%, but epidemiological changes having a significant negative impact (−262.02%). In the high‐income Asia‐Pacific region, aging contributed 66.52% to mortality, while epidemiological changes in high‐SDI regions had a contribution rate of 1188.54%. At the DALY level, the global loss was mainly attributed to population growth (258.83%), with aging and epidemiological changes contributing 3.35% and −155.48%, respectively. The DALY changes in Tropical Latin America were primarily driven by improvements in epidemiological changes (512.57%), while the DALYs in Eastern Europe increased due to worsening epidemiological factors (55.94%). These results indicate that population structure changes are the main driver of the increasing disease burden, but significant regional differences in epidemiological factors and aging effects substantially impact the disease burden distribution (Figure 6 and Supporting Information S1: Table 2).

### 3.7 Future Predictions

**Figure 7:**
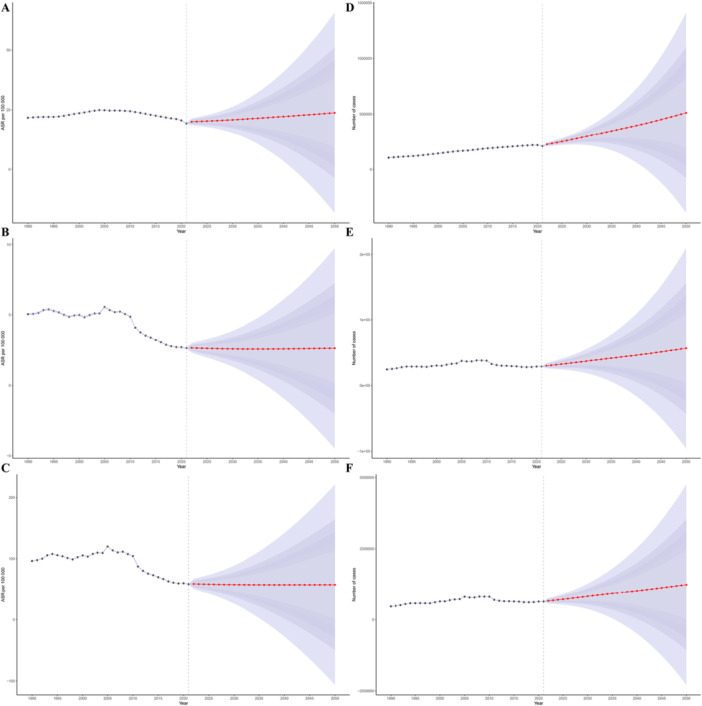
Bayesian age‐period‐cohort model projections of age‐standardized rates and absolute burden of alcoholic cardiomyopathy among adults aged ≥ 60 years, 2022–2050. (A–C) Projected age‐standardized prevalence rate (ASPR; A), mortality rate (ASMR; B), and DALY rate (ASDR; C) from 2022 to 2050. Solid lines represent point estimates; shaded areas indicate 95% confidence intervals. The vertical dashed line at 2022 marks the boundary between historical GBD estimates (1990–2021) and model projections (2022–2050). (D–F) Projected absolute number of prevalence cases (D), deaths (E), and DALYs (F) from 2022 to 2050. Unlike age‐standardized rates, absolute burden is projected to increase substantially over time due to population aging and growth. ASDR, age‐standardized disability‐adjusted life years rate (per 100,000 population); ASMR, age‐standardized mortality rate (per 100,000 population); ASPR, age‐standardized prevalence rate (per 100,000 population); BAPC, Bayesian age‐period‐cohort; CI, confidence interval; DALYs, disability‐adjusted life years; GBD, Global Burden of Disease; INLA, integrated nested Laplace approximation.

The ASPR is expected to rise slightly from 20.03 per 100,000 in 2022 to 23.75 per 100,000 in 2050; ASMR is projected to remain relatively stable, decreasing slightly from 2.67 per 100,000 in 2022 to 2.64 per 100,000 in 2050. Notably, although ASDR has continuously decreased from 1990 to 2021, it is expected to plateau during the forecast period, with an estimated value of 57.44 per 100,000 years in 2050. Overall, the change in ASR will be limited (18.6% increase in ASPR, 1.1% decrease in ASMR, and 1.8% decrease in ASDR), indicating that the global burden of alcoholic cardiomyopathy in the elderly population may reach a relatively stable phase (Figure 7A–C and Supporting Information S2: Table 3). However, the number of patients, deaths, and DALYs will continue to gradually increase over time (Figure 7D–F).

### 3.8 Attributable Risk Factors for ASDR

Risk factor analysis based on ASDR revealed that in 2021, the global disease burden of alcoholic cardiomyopathy among individuals aged 60 and older was primarily attributed to high alcohol use (58.53 per 100,000), significantly higher than temperature‐related risk factors (cold: 3.78 per 100,000 years; heat: 0.29 per 100,000 years). Notably, the impact of these risk factors exhibited significant heterogeneity across regions. In high‐SDI regions, the ASDR attributed to high alcohol use was 146.75 per 100,000 years, 20 times higher than that in low‐SDI regions (7.33 per 100,000 years). In the same regions, the burden of cold exposure (9.31 per 100,000 years) was the highest globally. In contrast, the attributable burden of these risk factors was lower in middle‐low SDI and low SDI regions (Supporting Information S1: Figure 3). From 1990 to 2021, the ASDR attributable to high alcohol use increased from 96.23 per 100,000 in 1990 to a peak of 119.98 per 100,000 years in 2005, before gradually decreasing to 58.53 per 100,000 years in 2021. The most significant reductions were observed in high‐SDI and middle‐high SDI regions, while low‐SDI regions maintained a relatively low level. Cold exposure had a significant impact, with global DALYs decreasing from 7.06 per 100,000 years in 1990 to 3.78 per 100,000 years in 2021, with the highest burden in middle‐high SDI regions (9.31 per 100,000 years in 2021). The impact of heat exposure remained relatively small (global values consistently below 0.60 per 100,000 years) (Supporting Information S1: Figure 4).

## 4 Discussion

Utilising data from GBD 2021, this study presents the first comprehensive evaluation of the global burden and trends of ACM among adults aged 60 and over from 1990 to 2021, with projections extending to 2050. The analysis reveals substantial heterogeneity in ACM burden across geographic and socioeconomic dimensions, influenced by demographic change, alcohol consumption patterns, and public health policies.

Globally, although age‐standardized rates (ASPR, ASMR, and ASDR) of ACM declined from 1990 to 2021, absolute case numbers, deaths, and DALYs increased, primarily driven by population growth and aging [29, 30]. The highest burden was reported in Eastern Europe, where national‐level disparities were markedly evident in countries such as Hungary, Russia, and Latvia. The existing evidence suggests that males consistently bear a disproportionate burden, with rates that are 3–5 times higher than those observed in females [31]. A parallel set of gender disparities is also evident in other alcohol‐related diseases, including alcoholic cirrhosis. This discrepancy in rates widened with age [32, 33, 34, 35]. Additionally, the relationship between the SDI and the ACM burden exhibited a non‐linear pattern, with an initial increase up to an approximate SDI of 0.6, followed by a subsequent decline. Projections indicate a stabilisation in age‐standardised mortality and DALY rates; however, a continued rise in the absolute burden is predicted due to demographic shifts.

Consistent with previous studies, our findings confirm an elevated incidence of ACM in Eastern Europe [36]. This phenomenon is likely attributable to culturally embedded drinking patterns, high consumption of spirits, and historical alcohol policies that promote affordability and availability [37, 38, 39]. Furthermore, the marked disparity observed in this study mirrors patterns seen in other alcohol‐related diseases, such as alcoholic cirrhosis [40], where biological differences, such as alcohol metabolism enzymes, elevated consumption levels among males, and gender‐specific drinking behaviours are significant contributing factors [41, 42]. The decline in age‐standardized rates is concomitant with enhanced diagnosis and management in high‐SDI regions; however, the rising absolute burden reflects a global demographic transition that has also been observed in other non‐communicable diseases [43, 44].

The elevated ACM burden in high‐ and middle‐high SDI countries may be linked to increased alcohol access during economic transition, coupled with insufficient public health responses [45, 46, 47]. The subsequent decline at very high SDI levels is likely to result from effective policy interventions, such as alcohol taxation, restrictions on marketing, and health promotion campaigns, which have been successfully implemented in countries like Germany [48, 49] and Austria [50, 51]. The persistent male preponderance may be indicative of two interrelated factors. Firstly, sociobehavioral norms that encourage heavy drinking among men [52, 53]. Secondly, physiological differences in ethanol metabolism [1, 3]. Moreover, the rise in absolute burden is largely attributable to population ageing, a trend compounded by longer life expectancies [54, 55] and increasing alcohol use among the elderly in many societies [56, 57].

These findings underscore the urgent necessity for targeted interventions in regions experiencing elevated disease burdens, such as Eastern Europe, and among high‐risk groups, notably older men. In regions experiencing high levels of burden, it is essential to strengthen health systems with a view to facilitating primary care‐based screening for alcohol use disorders and the delivery of brief interventions that have been tailored to meet the needs of older adults. Policy measures aimed at reducing the availability and appeal of high‐proof alcoholic beverages must be accorded the highest priority. Such measures may include the implementation of taxation schemes specifically targeting spirits, the restriction of marketing aimed at vulnerable populations, and the standardization of drink labeling requirements [58, 59]. Furthermore, there is an imperative for the development and dissemination of evidence‐based guidelines for alcohol consumption that are tailored to the elderly. It is imperative that these guidelines take into account age‐related changes in metabolism, polypharmacy, and comorbidities. Multi‐level interventions, combining health service enhancement with regulatory policy, have the potential to significantly mitigate the burden of ACM in aging populations. It is imperative to enhance diagnostic capacity in low‐SDI settings to reduce underdiagnosis and misclassification.

However, the present study is subject to several limitations. Firstly, reliance on GBD model estimates is associated with inherent uncertainties, including the redistribution of garbage codes and dependence on available vital registration data, which is sparse in many low‐SDI regions [12, 19, 60], and it should be noted that the GBD estimates rely on modeling and imputation for countries with limited primary data, which contributes to wider uncertainty intervals in low‐SDI regions. While the 95% UIs presented throughout this study capture this imputation uncertainty, the precision of estimates varies geographically based on underlying data availability. Secondly, the absence of individual‐level data hinders the capacity to adjust for key confounders, including genetic predispositions, specific beverage types, and drinking patterns [12]. Finally, it should be noted that the projections assume the continuation of current trends and do not take into account the possibility of future policy changes or public health crises. Our future research incorporates individual‐level epidemiological studies and integrates alcohol policy variables into burden forecasting models. In conclusion, this study highlights the growing absolute burden of ACM among older adults and its inequitable distribution across regions and sexes. Addressing this issue necessitates concerted efforts that integrate alcohol control policies with enhanced clinical capacity and targeted health promotion initiatives tailored to high‐risk populations.

## Author Contributions

**Siyi Xu:** conceptualization, methodology, software, data curation, investigation, visualization, writing – original draft, writing – review and editing. **Xuqing Ying:** visualization, software, and methodology. **Qian Hong:** methodology, software, and visualization. **Tingru Miao:** software, formal analysis, and data curation. **Weixun Cai:** writing – original draft, and methodology. **Yixin Chen:** writing – original draft. **Xin Chen:** software and writing – original draft. **Liuqin Hu:** writing – original draft, writing – review and editing, conceptualization, methodology, software, validation, visualization, formal analysis, supervision, and data curation.

## Funding

The authors have nothing to report.

## Conflicts of Interest

The authors declare no conflicts of interest. All ICMJE Disclosure Forms have been completed and submitted for each author, confirming that there are no financial or non‐financial relationships/activities/interests related to the content of this manuscript.

## Transparency Statement

The lead author Liuqin Hu affirms that this manuscript is an honest, accurate, and transparent account of the study being reported; that no important aspects of the study have been omitted; and that any discrepancies from the study as planned (and, if relevant, registered) have been explained.

## Supporting information

Supporting File 1

Supporting File 2

## Data Availability

The data that support the findings of this study are openly available in the Global Health Data Exchange (GHDx) repository, maintained by the Institute for Health Metrics and Evaluation (IHME), at http://ghdx.healthdata.org/gbd-results-tool. The data were accessed in August 2025. No new data were generated or analyzed beyond those publicly available from the GBD 2021 study.
