# Supplementary material for: Global Burden of Alcoholic Cardiomyopathy in Adults Aged 60 and Older From 1990 to 2021: A Secondary Analysis of Global Burden of Disease 2021 Data
Source: Health Sci Rep. 2026 Apr 14;9(4):e72358. doi: 10.1002/hsr2.72358 (PMC13079429; doi:10.1002/hsr2.72358)
Supplement: Supplementary file 1 — Supporting File 1 [file HSR2-9-e72358-s001.docx]

**Global Burden of Alcoholic Cardiomyopathy in Adults Aged 60 and Older from 1990 to 2021: A Secondary Analysis of Global Burden of Disease 2021 Data**

**Supplementary Figure 1**: Comparison of ASR in 1990 and 2021 for the Global, 5 SDI Regions, and 21 GBD Regions, along with their EAPC.

**Supplementary Figure 2**: Gender-Time and Age-Time Trend Analysis (A. ASPR; B. ASMR; C.ASDR; D. ASPR; E.ASMR; F.ASDR)

**Supplementary Figure 3**: Attributable Risk Factors for ASDR in 2021 for the Global and 5 SDI Regions.

**Supplementary Figure 4**: Temporal Trends of Attributable Risk Factors for ASDR in the Global and 5 SDI Regions.


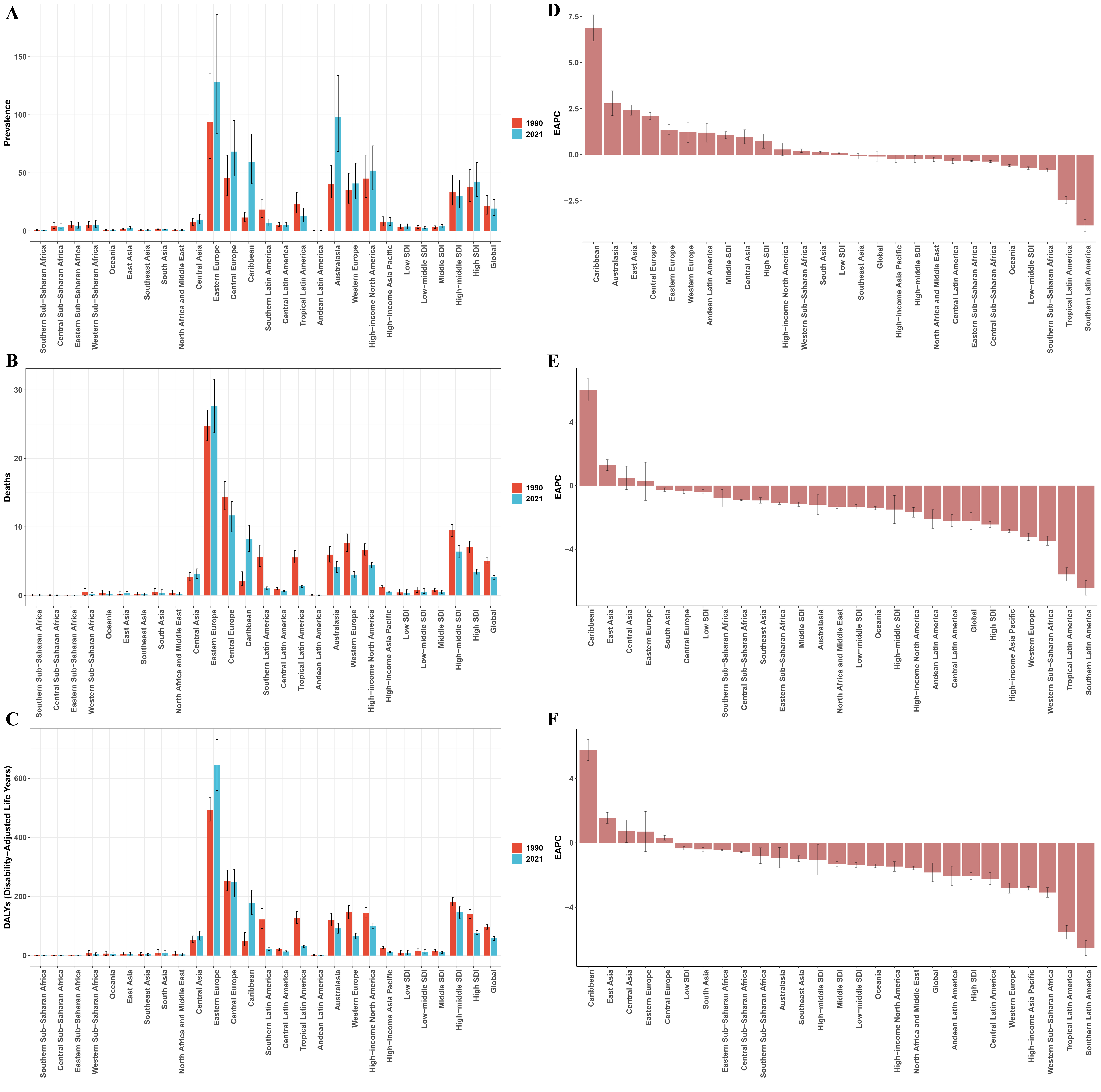


Supplementary Figure 1: Comparison of ASR in 1990 and 2021 for the Global, 5 SDI Regions, and 21 GBD Regions, along with their EAPC.


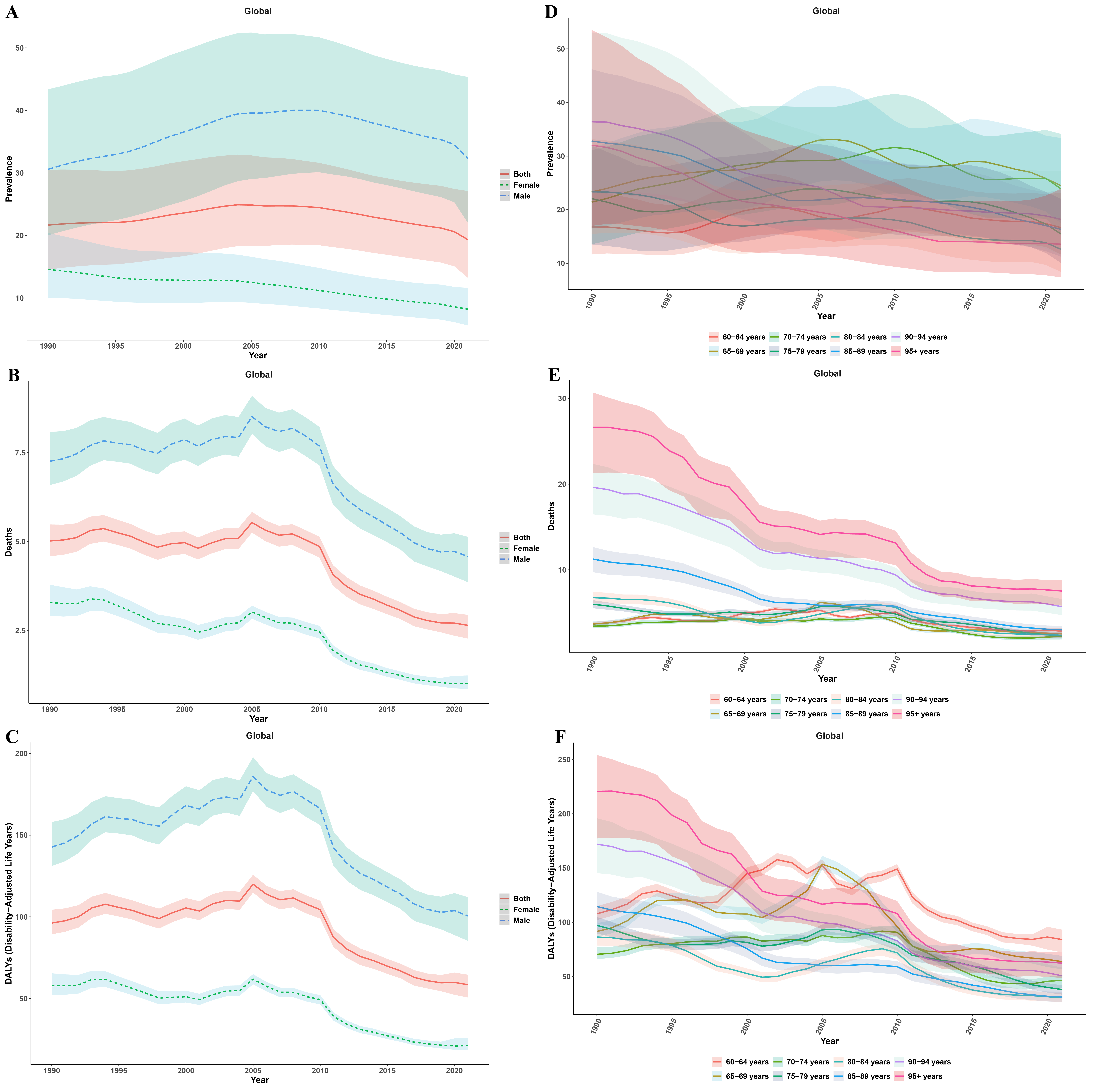


Supplementary Figure 2: Gender-Time and Age-Time Trend Analysis (A. ASPR; B. ASMR; C.ASDR; D. ASPR; E.ASMR; F.ASDR)


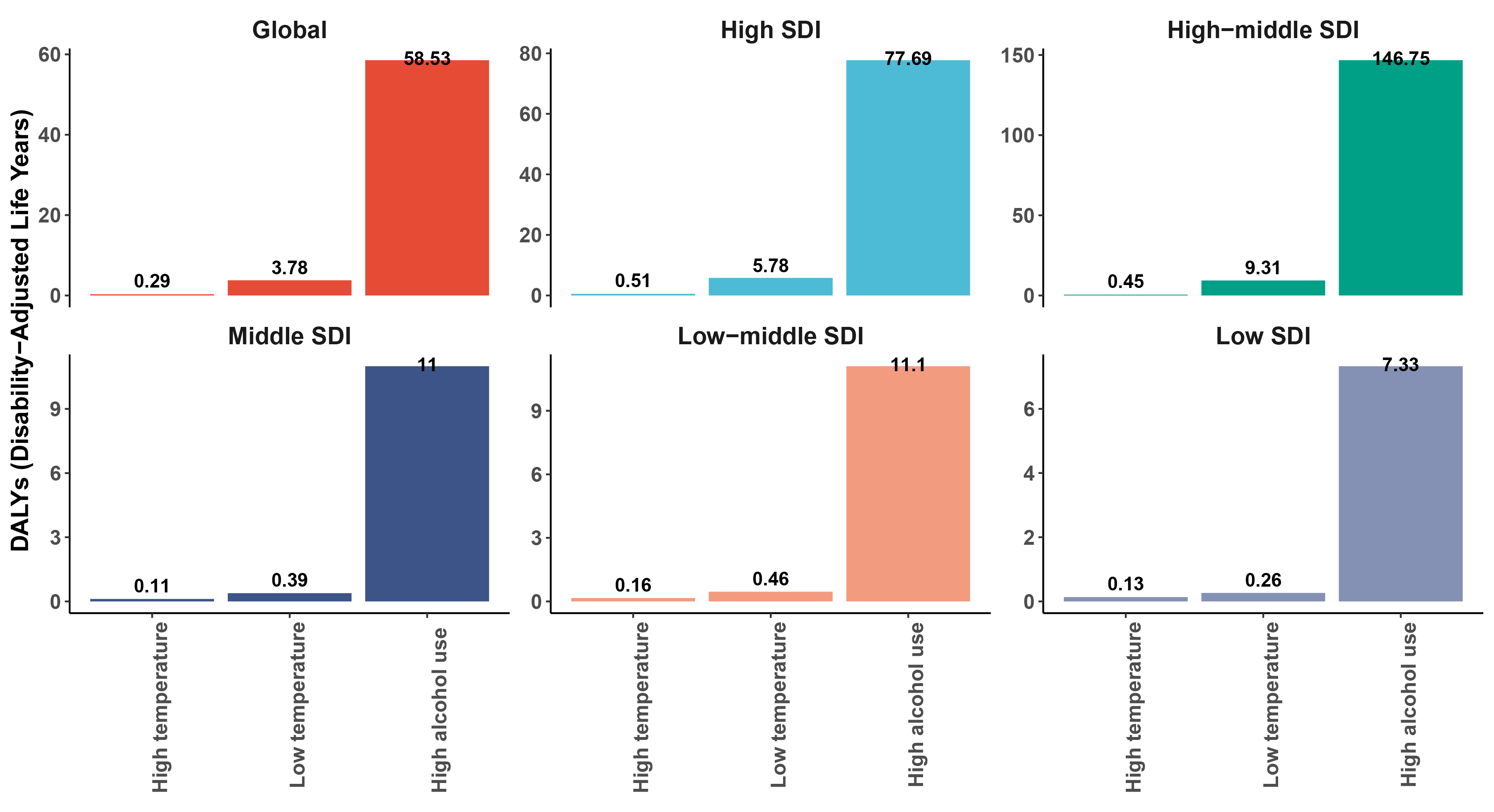


Supplementary Figure 3: Attributable Risk Factors for ASDR in 2021 for the Global and 5 SDI Regions.


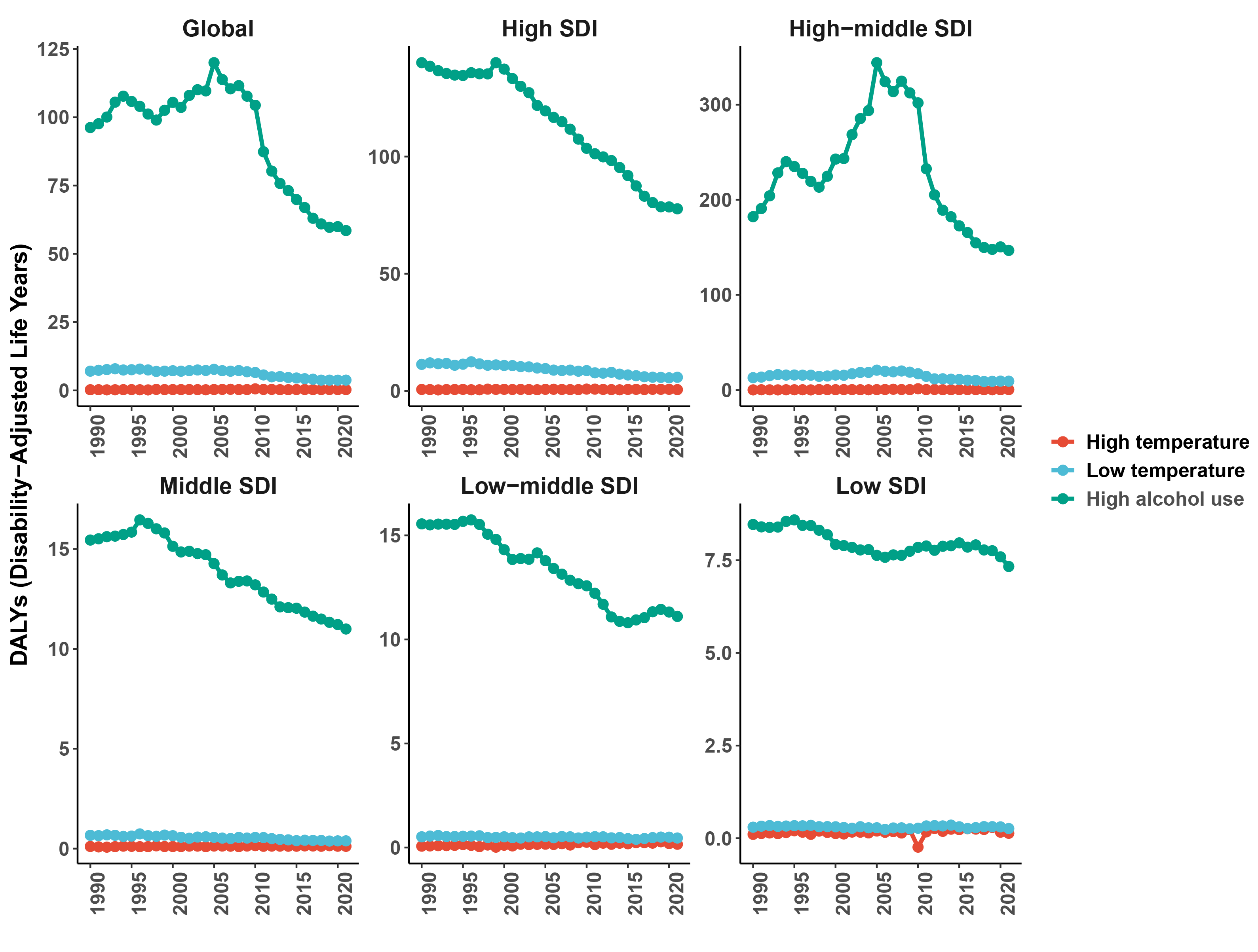


Supplementary Figure 4: Temporal Trends of Attributable Risk Factors for ASDR in the Global and 5 SDI Regions.
