# Supplementary material for: Global Burden of Alcoholic Cardiomyopathy in Adults Aged 60 and Older From 1990 to 2021: A Secondary Analysis of Global Burden of Disease 2021 Data
Source: Health Sci Rep. 2026 Apr 14;9(4):e72358. doi: 10.1002/hsr2.72358 (PMC13079429; doi:10.1002/hsr2.72358)
Supplement: Supplementary file 2 — Supporting File 2 [file HSR2-9-e72358-s002.docx]

**Global Burden of Alcoholic Cardiomyopathy in Adults Aged 60 and Older from 1990 to 2021: A Secondary Analysis of Global Burden of Disease 2021 Data**

**Supplementary Table 1**: **Age-standardized prevalence, mortality, and DALY rates of alcoholic cardiomyopathy with corresponding EAPC trends among adults aged ≥60 years in 204 countries and territories, 1990–2021.**

**Supplementary Table 2**: **Decomposition analysis of changes in alcoholic cardiomyopathy burden among adults aged ≥60 years, by region and SDI level, 1990–2021.**

**Supplementary Table 3**: **Bayesian age-period-cohort model projections of age-standardized rates of alcoholic cardiomyopathy among adults aged ≥60 years, 2022–2050.**

Supplementary Table 1: ASR and EAPCs for 204 Countries.

| location | ASPR | | | ASMR | | | ASDR | | |
| --- | --- | --- | --- | --- | --- | --- | --- | --- | --- |
|  | 1990 (per 100,000 population, 95% UI) | 2021 (per 100,000 population, 95% UI) | EAPCs (95% CI) | 1990 (per 100,000 population, 95% UI) | 2021 (per 100,000 population, 95% UI) | EAPCs (95% CI) | 1990 (per 100,000 population, 95% UI) | 2021 (per 100,000 population, 95% UI) | EAPCs (95% CI) |
| Afghanistan | 0.99(0.66,1.43) | 0.94(0.62,1.37) | 0.17(-0.32,0.66) | 0.61(0.09,1.96) | 0.50(0.08,1.72) | -0.51(-0.84,-0.19) | 12.37(1.90,38.21) | 9.90(1.67,34.03) | -0.59(-1.23,0.06) |
| Albania | 24.69(15.75,35.79) | 27.65(17.10,41.09) | -0.15(-0.95,0.66) | 6.06(1.62,11.25) | 4.15(0.81,8.90) | -1.45(-2.31,-0.58) | 104.77(32.08,191.08) | 75.32(15.90,157.75) | -1.42(-2.34,-0.49) |
| Algeria | 1.59(1.08,2.26) | 1.57(1.04,2.30) | 0.17(-0.52,0.86) | 0.55(0.07,1.67) | 0.41(0.08,1.15) | -0.35(-1.01,0.32) | 9.23(1.31,27.04) | 6.32(1.27,17.34) | -0.76(-1.06,-0.46) |
| American Samoa | 3.49(2.37,4.89) | 1.75(1.17,2.54) | -2.88(-3.34,-2.41) | 1.22(0.20,2.78) | 0.51(0.13,1.34) | -3.56(-4.10,-3.01) | 24.28(4.35,54.50) | 10.52(2.83,28.18) | -3.58(-4.19,-2.96) |
| Andorra | 51.28(33.99,73.50) | 52.97(34.15,77.33) | -0.05(-1.46,1.37) | 6.98(1.81,15.21) | 3.64(0.87,7.77) | -2.19(-3.12,-1.25) | 134.25(41.60,280.15) | 72.54(22.11,147.86) | -2.07(-3.15,-0.99) |
| Angola | 4.16(2.08,7.17) | 3.69(1.88,6.48) | 0.37(-2.12,2.92) | 0.02(0.00,0.09) | 0.01(0.00,0.06) | -1.00(-2.38,0.40) | 0.74(0.29,2.29) | 0.58(0.25,1.70) | -0.41(-2.34,1.56) |
| Antigua and Barbuda | 3.26(2.23,4.54) | 24.37(15.55,35.79) | 7.86(4.82,10.99) | 0.53(0.35,0.77) | 4.36(2.94,6.37) | 8.63(5.91,11.41) | 12.23(8.18,17.90) | 95.74(65.25,138.15) | 8.42(5.73,11.17) |
| Argentina | 16.74(10.39,24.79) | 4.49(2.67,6.76) | -4.83(-7.33,-2.27) | 5.44(3.63,7.69) | 0.76(0.53,1.04) | -6.78(-9.49,-3.99) | 120.24(80.81,168.84) | 15.83(11.13,21.61) | -6.97(-9.72,-4.13) |
| Armenia | 65.09(42.26,94.00) | 33.92(23.04,47.41) | -2.32(-2.84,-1.80) | 22.17(16.16,29.63) | 7.18(5.15,9.84) | -4.78(-5.48,-4.07) | 402.50(298.87,529.77) | 137.29(98.79,187.64) | -4.71(-5.46,-3.95) |
| Australia | 40.42(28.07,56.63) | 104.72(72.87,143.60) | 2.45(0.66,4.28) | 5.57(4.44,6.90) | 3.88(2.99,4.82) | -1.63(-3.46,0.24) | 109.84(89.61,133.94) | 87.55(69.40,107.69) | -1.18(-3.16,0.85) |
| Austria | 161.88(113.19,208.65) | 49.29(32.96,66.93) | -4.73(-5.72,-3.73) | 40.91(31.79,51.92) | 4.87(3.72,6.27) | -7.78(-9.01,-6.53) | 574.54(457.42,715.86) | 104.65(82.14,132.66) | -6.29(-7.54,-5.01) |
| Azerbaijan | 3.09(1.87,4.54) | 3.18(1.83,4.83) | -0.74(-1.95,0.48) | 1.26(0.11,3.60) | 1.03(0.09,3.17) | -1.76(-3.18,-0.31) | 27.73(2.68,80.24) | 22.70(2.20,71.13) | -1.81(-3.20,-0.40) |
| Bahamas | 17.00(11.46,24.13) | 50.26(32.55,74.01) | 4.49(2.23,6.80) | 4.81(3.29,6.77) | 13.67(9.94,18.81) | 4.62(2.54,6.76) | 110.30(75.89,154.60) | 299.93(219.78,411.11) | 4.45(2.40,6.54) |
| Bahrain | 3.94(2.71,5.59) | 4.49(3.06,6.40) | 0.79(0.60,0.97) | 1.84(0.29,3.91) | 1.06(0.18,2.42) | -2.36(-3.15,-1.56) | 31.14(5.44,64.14) | 16.54(3.50,36.92) | -2.65(-3.15,-2.15) |
| Bangladesh | 2.50(1.70,3.52) | 2.68(1.80,3.82) | 0.31(-1.57,2.23) | 0.68(0.11,2.05) | 0.49(0.07,1.51) | -0.86(-2.46,0.76) | 13.68(2.38,40.02) | 9.44(1.55,28.96) | -1.00(-2.74,0.78) |
| Barbados | 47.97(33.04,66.86) | 104.11(70.14,148.42) | 3.16(0.78,5.59) | 6.60(4.58,9.13) | 10.60(7.20,14.88) | 2.40(0.23,4.62) | 145.39(102.01,199.43) | 226.38(157.77,313.27) | 2.31(0.19,4.47) |
| Belarus | 61.52(40.36,87.96) | 45.12(30.03,65.00) | -1.47(-2.13,-0.81) | 16.79(12.48,22.66) | 8.87(6.64,11.68) | -2.99(-4.15,-1.81) | 374.98(282.95,496.66) | 222.21(168.68,290.21) | -2.59(-3.77,-1.40) |
| Belgium | 14.97(9.41,21.82) | 24.78(17.43,34.21) | 1.51(-0.07,3.11) | 3.07(2.34,3.96) | 2.60(1.93,3.38) | -1.93(-3.85,0.02) | 57.50(44.43,73.50) | 55.83(42.22,71.50) | -1.49(-3.49,0.56) |
| Belize | 9.30(6.32,13.15) | 49.82(32.30,73.38) | 7.22(4.79,9.71) | 1.24(0.64,2.01) | 7.60(5.67,9.96) | 7.97(5.83,10.16) | 28.38(15.29,45.23) | 168.19(127.29,218.21) | 7.82(5.74,9.95) |
| Benin | 4.83(2.45,8.44) | 5.35(2.76,9.12) | 1.11(-0.42,2.68) | 0.36(0.02,1.00) | 0.19(0.01,0.55) | -2.43(-2.58,-2.29) | 6.13(0.70,16.73) | 3.30(0.55,9.10) | -2.20(-3.15,-1.23) |
| Bermuda | 7.58(5.08,10.57) | 92.00(61.04,132.16) | 9.44(7.55,11.36) | 1.22(0.74,1.98) | 8.21(5.87,11.06) | 7.08(4.75,9.46) | 27.32(16.89,43.73) | 174.61(127.42,233.33) | 6.87(4.59,9.21) |
| Bhutan | 1.81(1.23,2.53) | 1.95(1.33,2.78) | 0.03(-1.72,1.81) | 0.48(0.08,1.66) | 0.40(0.06,1.25) | -0.69(-2.06,0.71) | 9.56(1.74,32.91) | 7.72(1.32,24.16) | -0.87(-2.36,0.64) |
| Bolivia (Plurinational State of) | 0.28(0.19,0.41) | 0.36(0.24,0.53) | 0.92(0.81,1.02) | 0.13(0.01,0.34) | 0.08(0.00,0.21) | -1.15(-1.48,-0.82) | 2.15(0.16,5.76) | 1.43(0.12,3.50) | -1.12(-1.53,-0.71) |
| Bosnia and Herzegovina | 41.03(25.24,60.93) | 46.26(27.50,69.68) | 0.31(-0.88,1.52) | 11.65(2.38,25.63) | 9.62(1.62,21.94) | -1.13(-2.40,0.15) | 213.11(49.19,448.77) | 188.60(34.62,423.80) | -0.94(-2.24,0.37) |
| Botswana | 1.21(0.62,2.14) | 0.82(0.42,1.42) | -0.86(-3.49,1.84) | 0.07(0.01,0.20) | 0.03(0.00,0.10) | -1.40(-2.82,0.03) | 0.74(0.21,2.03) | 0.34(0.10,1.02) | -1.41(-2.93,0.15) |
| Brazil | 23.57(15.77,33.69) | 13.12(8.34,19.70) | -2.40(-4.55,-0.21) | 5.67(4.83,6.67) | 1.35(1.19,1.52) | -5.61(-7.85,-3.32) | 129.80(111.34,152.37) | 31.85(28.19,35.53) | -5.59(-7.97,-3.14) |
| Brunei Darussalam | 6.82(2.72,11.64) | 5.81(2.25,10.31) | -0.69(-1.81,0.44) | 4.26(0.97,9.55) | 2.04(0.50,4.72) | -2.46(-3.43,-1.49) | 86.35(20.89,187.47) | 40.84(10.72,93.78) | -2.65(-3.77,-1.52) |
| Bulgaria | 3.16(2.01,4.64) | 2.99(1.82,4.55) | -0.79(-1.74,0.17) | 1.19(0.90,1.54) | 1.47(1.04,2.02) | -0.62(-1.69,0.46) | 20.41(15.69,26.05) | 28.83(20.18,39.82) | -0.52(-1.71,0.68) |
| Burkina Faso | 5.12(2.63,8.83) | 6.13(3.12,10.46) | 1.39(-0.10,2.91) | 0.60(0.04,1.83) | 0.34(0.02,0.98) | -1.98(-2.18,-1.78) | 10.23(1.06,30.71) | 5.66(0.77,16.05) | -1.86(-2.92,-0.79) |
| Burundi | 4.75(2.43,8.11) | 5.43(2.71,9.44) | 0.61(-3.28,4.66) | 0.01(0.00,0.03) | 0.00(0.00,0.03) | -0.93(-2.73,0.89) | 0.55(0.24,1.20) | 0.59(0.25,1.26) | -0.00(-3.22,3.33) |
| Cabo Verde | 6.43(3.23,11.04) | 6.47(3.34,11.23) | 0.08(-0.84,1.01) | 0.09(0.00,0.28) | 0.06(0.00,0.16) | -1.50(-2.73,-0.25) | 1.53(0.38,4.18) | 1.24(0.38,2.92) | -0.79(-1.10,-0.48) |
| Cambodia | 0.60(0.41,0.86) | 0.73(0.49,1.05) | 0.65(-0.91,2.24) | 0.20(0.04,0.53) | 0.20(0.03,0.49) | -0.01(-1.41,1.41) | 4.13(0.90,10.76) | 3.74(0.64,9.21) | -0.35(-1.95,1.28) |
| Cameroon | 5.50(2.80,9.45) | 5.81(2.96,10.01) | 0.83(-0.58,2.25) | 0.40(0.02,1.24) | 0.20(0.01,0.60) | -2.98(-3.16,-2.81) | 6.68(0.73,20.04) | 3.61(0.59,10.29) | -2.65(-3.62,-1.67) |
| Canada | 37.99(26.51,51.90) | 67.79(44.76,98.01) | 1.40(-0.08,2.91) | 3.82(2.85,5.02) | 2.61(2.05,3.29) | -1.74(-3.58,0.14) | 83.52(62.99,109.12) | 60.59(48.49,74.67) | -1.53(-3.33,0.30) |
| Central African Republic | 3.69(1.85,6.41) | 3.37(1.72,5.83) | 0.39(-1.92,2.76) | 0.02(0.00,0.08) | 0.01(0.00,0.08) | -0.73(-2.13,0.70) | 0.71(0.26,2.14) | 0.59(0.23,1.89) | -0.29(-2.13,1.58) |
| Chad | 4.62(2.34,7.94) | 5.86(2.99,10.09) | 1.24(-0.30,2.81) | 0.48(0.03,1.49) | 0.26(0.02,0.79) | -2.46(-2.67,-2.24) | 7.66(0.77,22.59) | 4.43(0.71,13.49) | -2.17(-3.04,-1.30) |
| Chile | 8.89(5.49,12.86) | 7.53(4.80,11.09) | -1.14(-3.79,1.58) | 1.98(1.44,2.64) | 0.65(0.45,0.89) | -3.32(-6.06,-0.50) | 42.70(31.40,56.86) | 14.40(10.22,19.67) | -3.29(-6.09,-0.41) |
| China | 1.20(0.78,1.77) | 2.45(1.48,3.79) | 3.01(1.68,4.36) | 0.19(0.06,0.45) | 0.31(0.05,0.53) | 1.79(0.69,2.89) | 3.74(1.34,8.67) | 6.00(1.12,9.84) | 1.79(0.53,3.06) |
| Colombia | 1.07(0.73,1.51) | 3.26(2.15,4.70) | 2.84(1.65,4.04) | 0.21(0.16,0.27) | 0.30(0.21,0.42) | -0.35(-1.62,0.95) | 4.40(3.36,5.62) | 6.49(4.51,8.97) | -0.28(-1.66,1.13) |
| Comoros | 4.74(2.38,8.22) | 4.13(2.09,7.13) | 0.02(-3.94,4.13) | 0.00(0.00,0.03) | 0.00(0.00,0.02) | -1.55(-3.22,0.15) | 0.53(0.24,1.12) | 0.44(0.19,0.86) | -0.49(-3.74,2.88) |
| Congo | 3.28(1.66,5.70) | 2.78(1.43,4.87) | -0.23(-2.47,2.08) | 0.01(0.00,0.08) | 0.01(0.00,0.06) | -1.09(-2.32,0.16) | 0.59(0.25,1.78) | 0.47(0.20,1.52) | -0.73(-2.47,1.03) |
| Cook Islands | 0.02(0.01,0.04) | 0.03(0.01,0.06) | -0.14(-0.71,0.43) | 0.00(0.00,0.01) | 0.00(0.00,0.00) | -4.63(-5.21,-4.05) | 0.06(0.00,0.16) | 0.02(0.00,0.06) | -4.19(-4.81,-3.57) |
| Costa Rica | 11.21(7.63,15.82) | 16.27(10.74,23.46) | 0.94(-1.24,3.16) | 1.71(1.17,2.40) | 1.44(1.02,1.99) | -1.40(-3.70,0.95) | 36.32(25.28,50.33) | 30.98(21.94,42.43) | -1.32(-3.74,1.16) |
| Croatia | 36.57(21.35,55.24) | 73.18(41.40,110.10) | 0.05(-0.91,1.01) | 12.99(10.57,15.72) | 19.40(15.10,24.33) | -2.07(-3.72,-0.40) | 193.32(158.98,231.67) | 378.79(293.19,478.60) | -1.08(-2.72,0.58) |
| Cuba | 9.56(6.64,13.32) | 111.31(75.48,157.44) | 9.64(6.67,12.69) | 1.21(0.85,1.65) | 14.47(11.25,18.20) | 10.25(7.41,13.15) | 26.61(19.03,35.74) | 311.20(246.55,387.44) | 10.15(7.41,12.96) |
| Cyprus | 9.15(5.51,13.49) | 10.53(6.80,15.45) | -0.36(-2.32,1.64) | 3.50(0.61,8.26) | 1.41(0.23,3.24) | -3.48(-5.19,-1.73) | 63.75(12.89,146.33) | 27.73(5.71,61.97) | -3.32(-5.23,-1.38) |
| Czechia | 4.93(3.05,7.31) | 21.63(12.94,32.97) | 4.33(2.10,6.61) | 1.02(0.77,1.30) | 2.32(1.60,3.21) | 1.09(-1.42,3.67) | 19.33(14.71,24.62) | 50.02(34.38,69.55) | 1.37(-1.24,4.05) |
| Cote d'Ivoire | 5.24(2.69,8.96) | 5.78(3.01,9.88) | 0.97(-0.46,2.42) | 0.46(0.02,1.28) | 0.21(0.01,0.59) | -2.95(-3.17,-2.74) | 7.81(0.83,22.36) | 3.79(0.64,10.28) | -2.69(-3.69,-1.68) |
| Democratic People's Republic of Korea | 1.97(1.35,2.82) | 2.61(1.74,3.79) | 0.94(0.22,1.67) | 0.55(0.13,1.54) | 0.61(0.13,1.72) | 0.56(0.02,1.11) | 9.36(2.58,24.19) | 10.54(2.62,27.17) | 0.56(-0.21,1.33) |
| Democratic Republic of the Congo | 4.20(2.09,7.27) | 3.65(1.85,6.28) | 0.26(-1.92,2.48) | 0.02(0.00,0.09) | 0.01(0.00,0.07) | -0.51(-1.87,0.87) | 0.70(0.28,2.09) | 0.59(0.23,1.80) | -0.13(-1.93,1.69) |
| Denmark | 13.22(8.23,18.94) | 27.97(17.30,42.28) | 2.24(-0.02,4.56) | 3.67(2.84,4.60) | 2.43(1.83,3.17) | -2.99(-5.17,-0.76) | 78.07(61.16,96.94) | 51.20(39.28,65.83) | -2.96(-5.25,-0.62) |
| Djibouti | 4.78(2.41,8.29) | 4.80(2.40,8.21) | 0.01(-3.95,4.14) | 0.00(0.00,0.03) | 0.00(0.00,0.02) | -0.83(-2.54,0.91) | 0.53(0.23,1.11) | 0.51(0.22,1.05) | -0.27(-3.53,3.10) |
| Dominica | 12.19(8.12,17.31) | 18.54(11.97,27.29) | 1.38(-1.72,4.57) | 3.49(0.87,8.92) | 4.36(0.67,10.70) | 0.79(-2.17,3.85) | 77.15(20.33,194.05) | 99.55(16.67,239.63) | 0.85(-2.10,3.89) |
| Dominican Republic | 9.31(6.44,12.94) | 17.19(10.96,25.31) | 2.27(-0.76,5.40) | 1.15(0.34,2.93) | 2.42(0.38,5.11) | 3.08(0.18,6.06) | 25.94(8.56,62.82) | 54.66(10.39,111.41) | 3.05(0.20,5.98) |
| Ecuador | 0.16(0.10,0.24) | 0.18(0.11,0.26) | 5.39(3.43,7.38) | 0.10(0.07,0.17) | 0.03(0.02,0.04) | 1.87(-1.11,4.94) | 1.82(1.15,2.92) | 0.42(0.27,0.65) | 1.75(-1.16,4.75) |
| Egypt | 0.05(0.02,0.09) | 0.07(0.03,0.12) | 0.31(-0.20,0.82) | 0.01(0.00,0.03) | 0.00(0.00,0.01) | -2.17(-2.35,-1.99) | 0.17(0.04,0.53) | 0.08(0.03,0.26) | -2.14(-2.52,-1.75) |
| El Salvador | 1.30(0.88,1.84) | 1.07(0.71,1.55) | -0.82(-3.10,1.50) | 0.15(0.02,0.40) | 0.10(0.01,0.25) | -1.53(-3.67,0.67) | 3.32(0.53,8.78) | 2.32(0.38,5.56) | -1.56(-3.83,0.77) |
| Equatorial Guinea | 3.65(1.84,6.33) | 2.08(1.06,3.60) | -1.82(-4.24,0.65) | 0.02(0.00,0.09) | 0.01(0.00,0.04) | -3.41(-4.99,-1.81) | 0.67(0.27,2.18) | 0.33(0.15,0.98) | -2.69(-4.71,-0.62) |
| Eritrea | 3.56(1.79,6.21) | 3.47(1.75,6.04) | 0.17(-3.67,4.15) | 0.00(0.00,0.02) | 0.00(0.00,0.02) | -0.24(-1.92,1.47) | 0.41(0.18,0.87) | 0.39(0.17,0.86) | -0.09(-3.17,3.09) |
| Estonia | 38.49(24.66,56.55) | 62.18(36.81,96.34) | 0.68(-0.76,2.15) | 11.30(8.86,14.22) | 14.82(11.57,18.62) | 0.08(-1.96,2.17) | 259.07(203.44,327.04) | 355.24(280.07,444.37) | 0.16(-1.91,2.27) |
| Eswatini | 1.03(0.52,1.82) | 0.69(0.36,1.20) | -0.74(-3.30,1.89) | 0.05(0.01,0.15) | 0.01(0.00,0.04) | -1.81(-3.20,-0.41) | 0.60(0.16,1.52) | 0.21(0.07,0.50) | -1.65(-3.11,-0.16) |
| Ethiopia | 5.23(2.82,8.74) | 5.46(2.97,9.10) | 0.87(-3.52,5.46) | 0.00(0.00,0.02) | 0.00(0.00,0.02) | -1.60(-3.38,0.21) | 0.57(0.27,1.10) | 0.56(0.27,1.08) | -0.08(-3.63,3.60) |
| Fiji | 0.17(0.11,0.24) | 0.18(0.12,0.26) | 0.11(-0.32,0.53) | 0.07(0.01,0.15) | 0.05(0.01,0.12) | -0.71(-1.64,0.22) | 1.41(0.19,3.07) | 1.14(0.14,2.51) | -0.69(-1.73,0.35) |
| Finland | 44.90(26.11,66.40) | 78.97(48.58,119.63) | 1.46(-0.00,2.95) | 13.51(10.37,16.96) | 9.48(7.38,11.81) | -2.16(-4.14,-0.14) | 288.73(223.04,359.92) | 209.16(166.11,257.09) | -1.86(-3.92,0.23) |
| France | 32.55(22.63,45.70) | 71.99(48.34,104.90) | 1.87(-0.03,3.80) | 2.25(1.79,2.78) | 1.83(1.31,2.47) | -1.56(-3.75,0.67) | 45.68(36.93,56.06) | 45.22(33.46,59.54) | -0.86(-3.14,1.47) |
| Gabon | 3.78(1.90,6.53) | 2.37(1.19,4.12) | -0.89(-3.24,1.52) | 0.01(0.00,0.08) | 0.01(0.00,0.05) | -1.68(-3.16,-0.17) | 0.63(0.27,1.85) | 0.39(0.17,1.22) | -1.30(-3.25,0.70) |
| Gambia | 4.90(2.50,8.44) | 5.28(2.73,9.16) | 1.03(-0.42,2.49) | 0.49(0.03,1.39) | 0.25(0.01,0.71) | -2.53(-2.78,-2.27) | 8.36(0.84,23.90) | 4.12(0.60,11.57) | -2.38(-3.36,-1.38) |
| Georgia | 4.37(2.54,6.57) | 2.99(1.14,5.10) | -4.42(-6.32,-2.47) | 1.37(0.96,2.04) | 1.11(0.77,1.53) | -4.22(-6.57,-1.81) | 28.65(20.27,41.48) | 25.65(17.53,35.58) | -3.94(-6.28,-1.55) |
| Germany | 93.37(60.43,134.37) | 81.32(51.84,119.78) | 0.37(-0.95,1.71) | 22.36(17.32,27.86) | 8.24(6.41,10.22) | -4.42(-6.05,-2.75) | 454.16(358.11,556.35) | 175.22(139.24,213.95) | -4.18(-5.79,-2.54) |
| Ghana | 5.34(2.74,9.16) | 4.65(2.34,8.05) | -0.57(-3.18,2.11) | 0.91(0.10,2.78) | 0.47(0.06,1.50) | -2.66(-3.91,-1.40) | 18.92(2.52,58.53) | 9.83(1.70,31.27) | -2.63(-4.83,-0.39) |
| Greece | 3.34(2.22,4.76) | 2.69(1.76,3.81) | -1.56(-2.84,-0.27) | 0.66(0.43,0.98) | 0.51(0.34,0.72) | -1.32(-2.46,-0.17) | 12.94(8.69,19.00) | 10.29(7.01,14.45) | -1.29(-2.63,0.06) |
| Greenland | 11.26(5.52,17.81) | 26.91(13.42,42.25) | 2.52(-0.87,6.02) | 3.02(0.59,7.19) | 3.59(0.36,8.10) | 0.09(-3.48,3.79) | 72.59(14.51,168.43) | 86.01(10.67,189.22) | 0.11(-3.42,3.77) |
| Grenada | 32.54(22.31,45.94) | 69.12(45.12,98.32) | 2.68(0.75,4.66) | 7.39(5.15,10.06) | 15.52(11.35,20.26) | 2.48(0.44,4.57) | 164.66(117.01,221.31) | 342.43(252.69,444.16) | 2.37(0.45,4.34) |
| Guam | 1.98(1.34,2.79) | 1.63(1.07,2.40) | -1.04(-2.07,0.00) | 0.80(0.17,1.71) | 0.30(0.06,0.68) | -3.75(-4.74,-2.76) | 16.41(3.81,34.99) | 6.98(1.56,15.57) | -3.49(-4.66,-2.31) |
| Guatemala | 3.70(2.51,5.30) | 3.33(2.19,4.83) | -0.98(-1.48,-0.48) | 0.57(0.37,0.84) | 0.32(0.23,0.44) | -2.44(-2.81,-2.07) | 10.67(7.01,15.34) | 6.59(4.68,9.01) | -2.18(-2.63,-1.73) |
| Guinea | 4.98(2.53,8.63) | 5.63(2.94,9.67) | 1.07(-0.45,2.61) | 0.37(0.02,1.16) | 0.22(0.01,0.64) | -2.06(-2.23,-1.88) | 6.20(0.69,19.67) | 3.82(0.61,10.54) | -1.87(-2.72,-1.01) |
| Guinea-Bissau | 4.63(2.36,8.07) | 4.98(2.57,8.63) | 0.97(-0.45,2.41) | 0.52(0.03,1.50) | 0.25(0.02,0.71) | -2.63(-2.82,-2.43) | 9.14(0.94,25.84) | 4.14(0.59,11.49) | -2.50(-3.54,-1.46) |
| Guyana | 9.25(6.17,13.34) | 34.17(21.86,50.37) | 5.68(3.65,7.74) | 5.16(3.56,7.25) | 12.81(8.17,19.08) | 5.48(3.69,7.31) | 117.19(81.42,163.47) | 289.93(186.51,428.31) | 5.40(3.65,7.17) |
| Haiti | 20.68(13.90,29.36) | 26.62(17.79,37.69) | 0.89(-1.63,3.48) | 6.74(2.14,16.05) | 7.03(1.81,17.19) | 0.27(-1.45,2.02) | 152.04(51.47,349.06) | 157.80(44.34,375.16) | 0.29(-1.36,1.97) |
| Honduras | 4.91(3.32,7.05) | 3.32(2.15,4.85) | -1.46(-3.06,0.17) | 1.03(0.18,2.54) | 0.98(0.14,2.40) | -0.19(-1.58,1.21) | 21.86(4.35,52.23) | 20.49(3.28,50.15) | -0.21(-1.73,1.33) |
| Hungary | 100.64(56.19,153.36) | 126.23(72.97,192.64) | 0.68(-0.50,1.87) | 46.25(36.62,57.58) | 35.87(28.49,44.24) | -1.18(-2.61,0.27) | 811.01(652.76,988.63) | 782.91(626.52,962.91) | -0.53(-1.96,0.93) |
| Iceland | 5.48(3.10,8.26) | 7.50(4.53,11.69) | -0.43(-2.26,1.43) | 2.03(1.52,2.61) | 0.92(0.66,1.23) | -3.43(-5.23,-1.59) | 39.20(29.61,50.16) | 17.92(13.03,23.86) | -3.52(-5.44,-1.56) |
| India | 1.60(1.06,2.34) | 1.64(0.99,2.50) | 0.27(-1.49,2.06) | 0.40(0.08,0.90) | 0.38(0.07,0.84) | 0.04(-1.55,1.65) | 8.58(1.91,19.04) | 7.71(1.47,17.20) | -0.19(-1.88,1.53) |
| Indonesia | 0.82(0.53,1.22) | 0.79(0.47,1.24) | -0.26(-1.69,1.20) | 0.19(0.03,0.41) | 0.23(0.03,0.48) | 0.65(-0.54,1.85) | 3.81(0.83,8.47) | 4.27(0.71,8.83) | 0.21(-1.25,1.68) |
| Iran (Islamic Republic of) | 1.40(0.93,2.06) | 1.24(0.76,1.89) | -0.08(-1.27,1.14) | 0.41(0.06,1.15) | 0.22(0.03,0.58) | -1.54(-1.94,-1.14) | 7.55(1.40,21.57) | 4.02(0.81,11.06) | -1.52(-2.36,-0.68) |
| Iraq | 0.14(0.09,0.20) | 0.11(0.07,0.17) | -1.03(-7.30,5.66) | 0.05(0.01,0.16) | 0.05(0.00,0.12) | -1.02(-8.13,6.64) | 0.88(0.10,2.57) | 0.69(0.07,1.81) | -1.21(-7.94,6.01) |
| Ireland | 20.03(12.55,29.45) | 32.80(21.24,48.14) | 1.42(0.34,2.52) | 7.68(5.90,9.85) | 2.55(1.78,3.46) | -3.84(-5.03,-2.63) | 131.13(102.22,166.03) | 53.12(38.04,71.43) | -3.32(-4.65,-1.97) |
| Israel | 1.99(1.22,2.94) | 4.23(2.67,6.28) | 1.76(0.10,3.46) | 0.47(0.34,0.63) | 0.25(0.18,0.36) | -3.25(-4.78,-1.69) | 9.19(6.69,12.18) | 5.45(3.90,7.52) | -3.03(-4.73,-1.30) |
| Italy | 5.05(2.92,7.81) | 6.75(4.21,10.08) | 0.08(-1.23,1.41) | 0.91(0.78,1.01) | 0.42(0.33,0.51) | -5.20(-6.75,-3.64) | 14.80(13.09,16.33) | 8.74(6.95,10.60) | -4.33(-5.99,-2.64) |
| Jamaica | 16.10(10.84,23.10) | 37.41(24.13,54.90) | 3.80(-0.10,7.85) | 2.33(1.60,3.34) | 5.14(3.26,7.48) | 3.66(-0.06,7.52) | 53.02(36.82,74.84) | 114.59(73.30,165.66) | 3.58(-0.09,7.39) |
| Japan | 9.00(5.00,14.16) | 10.32(6.27,15.64) | -0.09(-2.26,2.12) | 1.40(1.22,1.61) | 0.72(0.63,0.82) | -3.12(-5.57,-0.61) | 30.51(26.77,35.10) | 16.38(14.40,18.48) | -2.99(-5.56,-0.34) |
| Jordan | 0.15(0.10,0.22) | 0.12(0.08,0.18) | -0.89(-1.14,-0.64) | 0.05(0.00,0.12) | 0.03(0.00,0.06) | -3.12(-4.47,-1.75) | 0.93(0.08,2.01) | 0.42(0.05,0.91) | -3.34(-4.28,-2.39) |
| Kazakhstan | 0.50(0.30,0.74) | 8.06(4.76,12.21) | 11.22(9.52,12.95) | 0.15(0.08,0.26) | 2.80(1.41,5.21) | 11.88(9.48,14.33) | 3.23(1.75,5.76) | 66.24(33.22,124.56) | 12.15(9.71,14.65) |
| Kenya | 4.61(2.52,7.62) | 3.48(1.91,5.73) | -1.29(-5.40,2.99) | 0.00(0.00,0.02) | 0.00(0.00,0.02) | -0.20(-1.85,1.48) | 0.48(0.23,0.92) | 0.37(0.17,0.73) | -1.02(-4.43,2.51) |
| Kiribati | 2.33(1.58,3.33) | 2.00(1.36,2.87) | -0.52(-0.84,-0.20) | 0.87(0.15,2.26) | 0.77(0.13,1.93) | -0.69(-0.89,-0.49) | 16.92(3.16,41.86) | 14.96(2.71,36.70) | -0.74(-1.08,-0.39) |
| Kuwait | 5.58(3.79,7.95) | 3.17(2.07,4.66) | -0.49(-2.24,1.29) | 1.26(0.80,1.93) | 0.71(0.39,1.13) | -2.43(-4.53,-0.29) | 26.29(17.26,39.74) | 13.59(7.63,21.28) | -2.61(-4.72,-0.45) |
| Kyrgyzstan | 29.08(17.87,42.47) | 69.38(43.27,102.87) | 3.29(2.18,4.41) | 9.84(7.76,12.48) | 23.93(18.57,30.36) | 3.35(1.82,4.91) | 210.08(166.86,264.29) | 498.25(388.17,631.73) | 3.35(1.82,4.91) |
| Lao People's Democratic Republic | 0.51(0.35,0.73) | 0.64(0.43,0.93) | 0.67(-0.66,2.01) | 0.27(0.04,0.71) | 0.21(0.03,0.55) | -0.77(-1.87,0.35) | 5.39(1.07,14.21) | 3.96(0.68,10.01) | -1.10(-2.44,0.26) |
| Latvia | 56.18(36.01,81.81) | 130.97(81.82,195.20) | 3.29(2.37,4.23) | 14.83(11.66,18.28) | 29.74(22.53,37.82) | 2.70(1.12,4.30) | 322.61(253.84,396.23) | 725.83(553.17,918.96) | 3.10(1.49,4.73) |
| Lebanon | 0.15(0.10,0.21) | 0.16(0.11,0.24) | 0.02(-3.09,3.23) | 0.05(0.00,0.14) | 0.02(0.00,0.06) | -2.32(-6.37,1.91) | 0.70(0.08,2.15) | 0.34(0.04,0.85) | -2.62(-6.22,1.11) |
| Lesotho | 0.96(0.49,1.68) | 0.72(0.37,1.25) | -0.62(-3.08,1.91) | 0.05(0.01,0.16) | 0.02(0.00,0.05) | -1.52(-2.64,-0.39) | 0.59(0.15,1.59) | 0.25(0.08,0.58) | -1.27(-2.50,-0.02) |
| Liberia | 5.61(2.84,9.64) | 6.05(3.08,10.39) | 0.94(-0.50,2.40) | 0.40(0.02,1.22) | 0.21(0.01,0.62) | -2.67(-2.96,-2.39) | 6.85(0.76,20.66) | 3.66(0.60,10.17) | -2.40(-3.14,-1.65) |
| Libya | 1.71(1.16,2.45) | 1.07(0.70,1.58) | -1.34(-1.89,-0.79) | 0.31(0.04,0.89) | 0.24(0.05,0.68) | -0.65(-0.85,-0.44) | 5.71(0.83,15.90) | 4.24(0.96,11.58) | -0.83(-1.25,-0.40) |
| Lithuania | 29.28(19.25,42.38) | 63.72(40.27,93.04) | 2.42(1.59,3.25) | 7.03(5.52,8.78) | 9.90(7.55,12.62) | 1.07(-0.44,2.61) | 151.98(119.64,189.94) | 242.20(185.00,307.64) | 1.44(-0.12,3.03) |
| Luxembourg | 27.78(15.34,42.38) | 38.92(24.01,58.57) | -0.15(-1.59,1.30) | 6.71(5.12,8.62) | 3.10(2.23,4.11) | -3.58(-5.20,-1.93) | 126.88(97.72,161.02) | 61.30(44.79,79.99) | -3.59(-5.31,-1.84) |
| Madagascar | 5.34(2.70,9.21) | 4.84(2.46,8.44) | 0.09(-3.49,3.79) | 0.01(0.00,0.04) | 0.01(0.00,0.03) | -0.62(-2.39,1.20) | 0.63(0.28,1.50) | 0.56(0.24,1.30) | -0.20(-3.15,2.84) |
| Malawi | 4.16(2.12,7.14) | 3.73(1.91,6.50) | 0.33(-3.85,4.69) | 0.00(0.00,0.02) | 0.00(0.00,0.02) | -0.19(-2.11,1.77) | 0.46(0.21,0.95) | 0.41(0.18,0.88) | -0.04(-3.42,3.46) |
| Malaysia | 0.04(0.03,0.06) | 0.03(0.02,0.04) | -2.59(-5.48,0.39) | 0.01(0.00,0.02) | 0.00(0.00,0.01) | -4.76(-6.93,-2.54) | 0.17(0.01,0.45) | 0.07(0.01,0.19) | -4.99(-7.33,-2.59) |
| Maldives | 1.54(1.06,2.13) | 2.14(1.43,3.04) | 1.09(0.90,1.28) | 0.43(0.08,1.01) | 0.23(0.04,0.47) | -2.53(-2.89,-2.18) | 8.87(1.91,20.57) | 4.30(0.90,8.77) | -2.79(-3.07,-2.50) |
| Mali | 4.96(2.52,8.52) | 5.69(2.94,9.76) | 1.10(-0.56,2.80) | 0.35(0.02,1.10) | 0.13(0.01,0.43) | -3.32(-3.51,-3.13) | 5.72(0.65,17.26) | 2.65(0.52,7.84) | -2.75(-3.61,-1.89) |
| Malta | 10.00(6.61,14.32) | 10.11(6.52,15.18) | 0.19(-1.10,1.50) | 2.91(2.19,3.78) | 0.93(0.65,1.28) | -4.56(-5.96,-3.14) | 55.53(42.18,71.82) | 19.58(14.08,26.53) | -4.33(-5.84,-2.80) |
| Marshall Islands | 0.70(0.48,1.01) | 0.58(0.40,0.83) | -0.75(-1.20,-0.31) | 0.42(0.04,1.08) | 0.26(0.03,0.70) | -1.72(-1.99,-1.44) | 8.26(0.88,21.58) | 5.15(0.64,14.29) | -1.75(-2.22,-1.28) |
| Mauritania | 4.68(2.35,8.12) | 5.48(2.85,9.42) | 1.02(-0.44,2.50) | 0.38(0.02,1.08) | 0.17(0.01,0.47) | -3.01(-3.29,-2.73) | 6.21(0.69,17.17) | 3.07(0.53,8.31) | -2.57(-3.32,-1.82) |
| Mauritius | 4.42(2.95,6.30) | 3.51(2.31,4.98) | -4.89(-6.70,-3.05) | 1.24(0.94,1.59) | 0.73(0.50,1.04) | -8.31(-10.55,-6.00) | 26.56(20.16,33.86) | 16.55(11.24,23.42) | -8.28(-10.60,-5.90) |
| Mexico | 5.03(3.35,7.32) | 5.94(3.67,9.02) | 0.17(-1.39,1.76) | 0.60(0.54,0.69) | 0.67(0.55,0.79) | -0.36(-1.83,1.12) | 12.30(11.02,13.91) | 14.57(12.14,17.27) | -0.20(-1.82,1.44) |
| Micronesia (Federated States of) | 0.77(0.52,1.09) | 0.64(0.43,0.92) | -0.72(-1.16,-0.29) | 0.52(0.04,1.44) | 0.30(0.03,0.79) | -1.85(-2.16,-1.54) | 9.76(0.98,26.40) | 5.94(0.69,15.85) | -1.77(-2.28,-1.27) |
| Monaco | 95.86(64.42,136.74) | 83.97(55.02,122.71) | -0.55(-1.55,0.45) | 17.90(4.81,34.42) | 8.84(2.59,17.31) | -2.70(-3.73,-1.66) | 328.68(103.22,611.32) | 164.72(56.13,311.76) | -2.68(-3.82,-1.52) |
| Mongolia | 31.76(16.66,48.34) | 37.49(18.73,58.62) | 0.59(-0.09,1.27) | 14.81(5.49,29.24) | 12.14(3.25,23.02) | -1.45(-2.55,-0.34) | 301.60(122.58,580.66) | 270.38(74.97,494.07) | -1.12(-2.18,-0.04) |
| Montenegro | 102.81(68.26,147.11) | 82.92(53.80,120.07) | -1.13(-2.28,0.03) | 22.72(5.52,45.82) | 19.95(3.61,41.22) | -1.02(-2.35,0.33) | 430.30(119.17,839.39) | 391.25(78.61,783.64) | -0.96(-2.31,0.40) |
| Morocco | 1.41(0.95,2.01) | 1.29(0.84,1.89) | -0.15(-0.51,0.21) | 0.52(0.07,1.64) | 0.49(0.09,1.43) | -0.16(-0.43,0.11) | 9.30(1.44,27.88) | 8.09(1.56,22.99) | -0.32(-0.51,-0.13) |
| Mozambique | 4.77(2.42,8.28) | 4.44(2.23,7.83) | -0.06(-2.36,2.30) | 0.00(0.00,0.02) | 0.00(0.00,0.02) | 0.10(-1.91,2.16) | 0.51(0.23,1.01) | 0.48(0.22,1.01) | -0.02(-2.30,2.30) |
| Myanmar | 0.69(0.47,0.97) | 0.78(0.52,1.12) | 0.44(-1.10,2.01) | 0.29(0.05,0.78) | 0.23(0.03,0.56) | -0.98(-2.25,0.31) | 5.75(1.16,15.58) | 4.27(0.77,10.76) | -1.24(-2.74,0.29) |
| Namibia | 1.13(0.57,1.99) | 0.94(0.48,1.64) | -0.13(-2.59,2.39) | 0.06(0.01,0.17) | 0.03(0.01,0.09) | -1.47(-2.79,-0.12) | 0.70(0.20,1.76) | 0.40(0.13,1.01) | -1.31(-2.75,0.14) |
| Nauru | 0.67(0.45,0.95) | 0.63(0.42,0.91) | 0.16(-0.12,0.43) | 0.47(0.05,1.29) | 0.43(0.04,1.11) | 0.22(-0.14,0.58) | 9.93(1.09,27.16) | 7.81(0.81,19.80) | -0.32(-0.81,0.17) |
| Nepal | 1.84(1.25,2.63) | 1.69(1.14,2.44) | -0.34(-1.86,1.20) | 0.52(0.09,1.59) | 0.42(0.07,1.16) | -0.62(-1.91,0.69) | 10.51(1.97,32.28) | 8.21(1.61,22.64) | -0.77(-2.19,0.66) |
| Netherlands | 40.28(28.91,55.14) | 42.88(27.60,63.07) | -0.46(-1.82,0.92) | 9.95(7.88,12.25) | 2.66(1.94,3.45) | -5.46(-6.99,-3.91) | 167.60(135.56,202.95) | 54.24(40.65,69.28) | -4.93(-6.53,-3.31) |
| New Zealand | 42.10(26.32,63.67) | 63.92(40.65,96.00) | 1.12(-0.52,2.79) | 7.87(5.87,10.55) | 5.33(4.27,6.55) | -1.82(-3.80,0.21) | 172.50(129.12,231.09) | 115.26(94.11,140.00) | -1.88(-3.96,0.24) |
| Nicaragua | 4.13(2.81,5.88) | 3.91(2.57,5.66) | -0.37(-2.43,1.73) | 0.50(0.08,1.12) | 0.40(0.06,0.89) | -0.82(-2.87,1.28) | 10.90(2.25,24.16) | 8.76(1.71,19.24) | -0.81(-2.96,1.38) |
| Niger | 4.99(2.55,8.53) | 5.42(2.82,9.29) | 1.03(-0.54,2.63) | 0.57(0.03,1.99) | 0.25(0.01,0.81) | -2.42(-2.72,-2.12) | 8.91(0.83,29.34) | 4.13(0.56,12.92) | -2.21(-2.95,-1.45) |
| Nigeria | 4.80(2.56,8.07) | 5.06(2.75,8.44) | 0.64(-0.76,2.07) | 0.50(0.02,1.01) | 0.14(0.01,0.30) | -4.33(-4.80,-3.85) | 7.30(0.75,15.30) | 2.51(0.47,5.69) | -3.86(-4.48,-3.23) |
| Niue | 0.75(0.52,1.06) | 0.58(0.39,0.82) | -1.04(-1.44,-0.64) | 0.34(0.03,0.84) | 0.20(0.02,0.48) | -2.13(-2.62,-1.64) | 6.67(0.68,16.41) | 4.03(0.50,9.80) | -2.15(-2.84,-1.45) |
| North Macedonia | 43.05(27.21,62.96) | 41.01(25.48,61.01) | -0.69(-1.95,0.59) | 16.97(3.70,34.92) | 11.58(1.99,26.73) | -2.05(-3.29,-0.80) | 298.70(71.19,604.89) | 218.02(39.17,506.04) | -1.94(-3.18,-0.69) |
| Northern Mariana Islands | 4.48(3.08,6.24) | 2.21(1.49,3.17) | -2.21(-3.50,-0.91) | 0.95(0.15,2.16) | 0.59(0.13,1.76) | -2.05(-3.16,-0.92) | 20.80(3.75,46.76) | 12.75(2.94,39.28) | -2.20(-3.51,-0.86) |
| Norway | 7.90(3.86,12.69) | 15.86(9.69,24.25) | 2.64(1.24,4.06) | 1.68(1.54,1.84) | 0.85(0.73,0.99) | -1.80(-3.58,0.01) | 40.38(37.06,43.98) | 21.10(18.24,24.37) | -1.66(-3.39,0.10) |
| Oman | 0.24(0.17,0.34) | 0.19(0.12,0.28) | -1.06(-1.79,-0.32) | 0.09(0.02,0.27) | 0.04(0.01,0.15) | -2.39(-2.95,-1.83) | 1.77(0.32,5.13) | 0.91(0.14,3.11) | -2.56(-3.44,-1.67) |
| Pakistan | 1.89(1.25,2.77) | 1.64(1.01,2.51) | -0.33(-2.08,1.46) | 0.47(0.09,1.32) | 0.43(0.07,1.17) | -0.41(-1.75,0.94) | 9.48(1.95,27.18) | 8.46(1.63,22.72) | -0.56(-2.03,0.93) |
| Palau | 0.64(0.45,0.91) | 0.53(0.36,0.75) | -0.88(-1.35,-0.40) | 0.23(0.02,0.57) | 0.14(0.01,0.35) | -1.61(-1.96,-1.27) | 4.48(0.47,11.27) | 2.82(0.33,6.98) | -1.72(-2.33,-1.10) |
| Palestine | 0.92(0.61,1.33) | 0.90(0.59,1.33) | -0.20(-1.12,0.74) | 0.47(0.05,1.22) | 0.32(0.03,0.76) | -1.74(-3.25,-0.20) | 6.80(0.89,17.52) | 4.58(0.63,10.80) | -1.74(-2.85,-0.61) |
| Panama | 6.57(4.46,9.34) | 7.00(4.73,10.08) | -0.55(-3.12,2.09) | 0.75(0.50,1.09) | 0.57(0.35,0.85) | -1.81(-4.57,1.03) | 16.14(10.86,23.18) | 12.50(7.90,18.29) | -1.74(-4.62,1.21) |
| Papua New Guinea | 0.89(0.62,1.25) | 0.80(0.55,1.11) | -0.61(-1.31,0.08) | 0.31(0.03,0.83) | 0.22(0.03,0.67) | -1.30(-1.76,-0.85) | 6.52(0.78,17.18) | 4.60(0.63,13.87) | -1.45(-2.04,-0.86) |
| Paraguay | 4.66(3.17,6.52) | 3.07(1.99,4.47) | -2.10(-4.66,0.53) | 0.69(0.08,1.56) | 0.42(0.08,0.98) | -2.44(-5.00,0.20) | 16.15(2.34,35.82) | 9.77(2.05,22.53) | -2.47(-5.20,0.33) |
| Peru | 0.39(0.26,0.55) | 0.41(0.26,0.59) | 0.59(-0.06,1.24) | 0.07(0.01,0.17) | 0.03(0.01,0.08) | -2.97(-3.65,-2.27) | 1.17(0.18,2.78) | 0.55(0.13,1.23) | -2.69(-3.30,-2.08) |
| Philippines | 1.12(0.73,1.66) | 0.97(0.58,1.50) | -0.12(-1.58,1.36) | 0.34(0.05,0.85) | 0.26(0.04,0.62) | -0.15(-1.25,0.96) | 5.88(1.22,16.03) | 4.83(1.05,12.44) | -0.30(-1.81,1.23) |
| Poland | 74.94(50.53,107.77) | 113.60(76.96,162.09) | 1.83(0.21,3.48) | 15.09(13.52,16.60) | 11.30(9.47,13.17) | -1.15(-3.20,0.94) | 262.57(237.35,287.21) | 252.43(211.78,294.01) | -0.33(-2.37,1.75) |
| Portugal | 11.64(7.92,16.55) | 20.53(13.42,29.85) | 0.81(-1.88,3.58) | 2.02(1.58,2.54) | 1.42(0.99,1.97) | -2.74(-5.20,-0.21) | 38.45(30.29,47.79) | 30.93(21.82,42.07) | -2.38(-5.04,0.35) |
| Puerto Rico | 7.87(5.41,11.03) | 26.14(17.24,37.82) | 5.28(2.26,8.39) | 0.88(0.61,1.23) | 1.85(1.31,2.52) | 3.17(0.05,6.38) | 19.94(14.04,27.48) | 42.40(30.62,56.65) | 3.19(0.11,6.37) |
| Qatar | 1.26(0.86,1.81) | 2.44(1.64,3.51) | 2.15(1.91,2.38) | 0.55(0.07,1.38) | 0.31(0.04,0.91) | -2.49(-3.27,-1.70) | 9.53(1.34,23.42) | 5.51(0.93,17.64) | -2.20(-2.84,-1.55) |
| Republic of Korea | 0.54(0.28,0.85) | 1.15(0.75,1.66) | 1.30(-2.08,4.79) | 0.09(0.01,0.21) | 0.04(0.00,0.13) | -3.96(-6.88,-0.95) | 1.96(0.29,4.49) | 1.03(0.18,2.90) | -3.74(-6.93,-0.44) |
| Republic of Moldova | 12.47(8.09,17.78) | 39.22(24.87,58.64) | 4.39(3.37,5.42) | 3.66(2.89,4.52) | 10.88(8.27,13.84) | 3.81(2.59,5.05) | 81.81(65.23,99.92) | 276.10(211.84,349.01) | 4.31(3.07,5.57) |
| Romania | 20.05(11.77,30.33) | 29.26(16.11,45.06) | 2.08(0.58,3.61) | 9.04(6.25,12.86) | 9.13(6.58,12.27) | -0.13(-1.34,1.10) | 176.66(121.53,251.55) | 194.12(139.99,261.88) | 0.13(-1.23,1.50) |
| Russian Federation | 104.67(68.98,152.36) | 149.46(97.85,217.29) | 1.57(0.95,2.18) | 24.72(23.26,25.87) | 29.36(25.22,33.16) | 0.09(-1.24,1.44) | 493.88(471.69,512.91) | 716.63(616.04,809.50) | 0.61(-0.77,2.00) |
| Rwanda | 4.67(2.37,7.98) | 4.38(2.21,7.54) | 0.21(-3.24,3.77) | 0.00(0.00,0.02) | 0.00(0.00,0.02) | -2.11(-3.74,-0.45) | 0.53(0.24,1.08) | 0.46(0.21,0.91) | -0.49(-3.43,2.55) |
| Saint Kitts and Nevis | 6.30(4.28,8.90) | 43.10(28.86,62.27) | 7.54(5.96,9.14) | 1.57(1.08,2.24) | 9.19(5.97,14.27) | 7.65(6.08,9.25) | 35.68(24.57,50.45) | 201.12(132.02,311.49) | 7.48(5.94,9.05) |
| Saint Lucia | 24.20(16.48,33.77) | 129.23(84.88,186.18) | 6.31(4.03,8.63) | 5.07(3.47,6.98) | 21.48(15.42,28.74) | 5.58(3.44,7.76) | 110.43(75.66,152.10) | 453.12(328.53,602.17) | 5.42(3.35,7.53) |
| Saint Vincent and the Grenadines | 13.04(8.76,18.43) | 45.38(29.16,66.48) | 4.47(2.18,6.81) | 2.55(1.95,3.30) | 9.60(7.36,12.11) | 4.21(2.25,6.21) | 55.56(43.15,70.96) | 197.99(152.95,247.12) | 3.99(2.10,5.92) |
| Samoa | 0.87(0.60,1.23) | 0.70(0.48,1.00) | -0.94(-1.44,-0.44) | 0.38(0.03,0.93) | 0.23(0.03,0.56) | -1.91(-2.32,-1.50) | 7.61(0.80,18.54) | 4.67(0.59,11.16) | -1.98(-2.58,-1.37) |
| San Marino | 27.92(18.61,39.55) | 36.05(23.46,52.58) | 0.33(-1.32,2.00) | 3.35(0.79,7.55) | 1.75(0.37,4.04) | -1.97(-3.57,-0.34) | 65.46(18.59,144.84) | 37.02(10.58,81.23) | -1.83(-3.56,-0.07) |
| Sao Tome and Principe | 4.90(2.48,8.46) | 4.80(2.51,8.34) | -0.12(-1.82,1.61) | 0.57(0.02,1.73) | 0.19(0.01,0.57) | -3.18(-3.77,-2.58) | 7.07(0.58,21.20) | 3.06(0.48,8.61) | -2.70(-3.33,-2.06) |
| Saudi Arabia | 1.45(0.97,2.09) | 0.99(0.65,1.45) | -1.36(-2.69,-0.00) | 0.67(0.10,1.80) | 0.35(0.06,0.87) | -2.44(-4.22,-0.62) | 11.07(1.70,28.13) | 5.70(1.09,13.92) | -2.49(-3.94,-1.03) |
| Senegal | 4.55(2.35,7.83) | 4.86(2.51,8.26) | 0.97(-0.54,2.50) | 0.42(0.02,1.28) | 0.20(0.01,0.62) | -2.66(-2.83,-2.49) | 7.52(0.83,22.30) | 3.56(0.57,10.14) | -2.48(-3.50,-1.45) |
| Serbia | 32.79(16.66,50.99) | 43.95(23.90,66.80) | -0.38(-2.66,1.95) | 18.35(5.43,39.87) | 13.94(2.62,28.87) | -1.76(-4.04,0.57) | 318.17(98.70,657.02) | 275.82(55.38,549.92) | -1.31(-3.69,1.14) |
| Seychelles | 0.36(0.24,0.51) | 0.54(0.35,0.78) | 1.02(-1.39,3.49) | 0.16(0.02,0.38) | 0.13(0.02,0.33) | -0.76(-2.72,1.24) | 3.17(0.58,7.77) | 2.54(0.37,6.51) | -0.96(-3.27,1.41) |
| Sierra Leone | 4.97(2.54,8.48) | 5.68(2.94,9.70) | 1.04(-0.39,2.48) | 0.47(0.03,1.46) | 0.21(0.01,0.62) | -3.04(-3.20,-2.88) | 8.12(0.83,24.64) | 3.73(0.60,10.85) | -2.75(-3.62,-1.87) |
| Singapore | 1.69(0.83,2.72) | 1.46(0.88,2.25) | -1.66(-3.61,0.32) | 0.94(0.69,1.24) | 0.13(0.09,0.19) | -6.96(-9.13,-4.75) | 19.41(14.25,25.74) | 2.81(1.95,3.93) | -7.04(-9.35,-4.68) |
| Slovakia | 10.60(7.01,15.11) | 30.01(19.28,44.16) | 3.64(1.73,5.58) | 3.20(1.14,6.22) | 5.20(0.94,9.60) | 1.21(-1.03,3.50) | 63.01(23.95,119.39) | 114.06(22.00,204.32) | 1.54(-0.82,3.97) |
| Slovenia | 70.50(40.35,106.42) | 80.83(48.47,119.69) | 0.94(0.71,1.18) | 22.34(13.02,35.99) | 8.78(6.11,12.33) | -3.01(-3.28,-2.74) | 402.77(243.06,624.82) | 153.71(109.10,214.15) | -3.23(-3.57,-2.89) |
| Solomon Islands | 0.68(0.46,0.96) | 0.60(0.41,0.87) | -0.38(-0.90,0.15) | 0.34(0.03,0.95) | 0.27(0.03,0.75) | -0.90(-1.33,-0.47) | 6.75(0.63,18.63) | 5.08(0.61,13.99) | -1.02(-1.65,-0.38) |
| Somalia | 4.17(2.12,7.15) | 3.48(1.76,6.02) | 0.11(-3.84,4.23) | 0.01(0.00,0.03) | 0.00(0.00,0.02) | -0.70(-2.64,1.28) | 0.50(0.22,1.06) | 0.41(0.17,0.91) | -0.29(-3.49,3.03) |
| South Africa | 0.50(0.28,0.84) | 0.41(0.23,0.69) | -0.40(-1.11,0.31) | 0.06(0.01,0.10) | 0.04(0.01,0.08) | -1.27(-2.19,-0.35) | 0.60(0.11,0.98) | 0.46(0.09,0.79) | -1.13(-1.90,-0.35) |
| South Sudan | 5.85(2.90,10.13) | 5.14(2.63,8.85) | 0.17(-4.45,5.03) | 0.01(0.00,0.04) | 0.00(0.00,0.02) | -1.35(-3.29,0.64) | 0.66(0.29,1.42) | 0.55(0.25,1.09) | -0.34(-3.99,3.45) |
| Spain | 16.63(10.87,23.81) | 21.82(15.86,28.66) | -0.24(-2.00,1.55) | 3.77(2.88,4.79) | 1.39(0.95,1.94) | -4.45(-6.37,-2.49) | 57.99(45.39,72.68) | 31.25(21.88,43.11) | -3.41(-5.49,-1.29) |
| Sri Lanka | 3.64(2.49,5.15) | 1.77(1.21,2.50) | -3.13(-4.95,-1.28) | 0.99(0.15,2.59) | 0.28(0.05,0.70) | -5.23(-7.10,-3.31) | 20.07(3.49,53.93) | 5.85(1.19,14.60) | -5.29(-7.26,-3.28) |
| Sudan | 1.30(0.88,1.84) | 1.01(0.67,1.50) | -0.62(-1.01,-0.22) | 0.55(0.08,1.76) | 0.36(0.07,1.08) | -1.59(-1.81,-1.36) | 10.41(1.62,30.97) | 6.30(1.39,19.23) | -1.77(-2.04,-1.49) |
| Suriname | 6.88(4.58,9.68) | 15.70(10.41,22.74) | 3.52(0.72,6.40) | 1.23(0.36,3.33) | 2.19(0.30,5.07) | 2.88(0.37,5.44) | 28.05(8.84,73.45) | 50.78(8.32,113.64) | 2.84(0.37,5.38) |
| Sweden | 23.26(15.20,33.98) | 71.85(45.03,110.17) | 3.46(1.57,5.38) | 1.08(0.85,1.34) | 2.53(1.88,3.30) | 1.56(-0.71,3.88) | 26.74(21.44,32.77) | 63.78(48.96,81.21) | 1.74(-0.45,3.98) |
| Switzerland | 31.73(18.23,47.14) | 36.65(22.86,54.58) | -0.16(-2.22,1.95) | 7.47(5.69,9.61) | 3.01(2.13,4.02) | -4.64(-6.86,-2.37) | 155.60(119.79,198.32) | 62.10(45.24,81.78) | -4.79(-7.13,-2.40) |
| Syrian Arab Republic | 1.39(0.94,1.98) | 1.13(0.74,1.66) | -0.58(-1.17,0.02) | 0.56(0.09,1.57) | 0.38(0.08,1.04) | -1.39(-1.84,-0.94) | 10.11(1.64,27.06) | 6.88(1.52,18.61) | -1.45(-1.75,-1.15) |
| Taiwan | 16.02(10.82,23.07) | 9.59(6.47,13.73) | -2.26(-2.93,-1.60) | 2.19(1.69,2.80) | 0.59(0.42,0.79) | -5.41(-6.19,-4.62) | 39.61(31.26,49.74) | 12.00(8.85,15.83) | -5.11(-6.02,-4.19) |
| Tajikistan | 0.05(0.03,0.09) | 0.07(0.03,0.11) | -0.35(-0.74,0.04) | 0.00(0.00,0.01) | 0.00(0.00,0.01) | -1.99(-4.03,0.09) | 0.08(0.01,0.20) | 0.05(0.01,0.14) | -1.65(-3.50,0.23) |
| Thailand | 0.55(0.38,0.78) | 1.32(0.90,1.87) | 2.08(0.27,3.94) | 0.06(0.01,0.15) | 0.08(0.01,0.20) | -0.23(-1.82,1.38) | 1.26(0.35,3.14) | 1.84(0.26,4.40) | 0.11(-1.61,1.85) |
| Timor-Leste | 0.87(0.60,1.23) | 0.85(0.57,1.23) | 0.04(-1.58,1.68) | 0.23(0.04,0.67) | 0.23(0.03,0.63) | 0.29(-0.79,1.38) | 4.64(0.88,13.47) | 4.34(0.75,11.86) | 0.04(-1.28,1.37) |
| Togo | 4.87(2.52,8.40) | 5.04(2.59,8.64) | 0.84(-0.49,2.18) | 0.45(0.02,1.36) | 0.25(0.01,0.68) | -2.22(-2.36,-2.07) | 7.54(0.78,21.85) | 3.92(0.57,10.52) | -2.10(-3.11,-1.08) |
| Tokelau | 0.93(0.65,1.29) | 0.78(0.54,1.11) | -0.47(-1.08,0.13) | 0.44(0.04,1.21) | 0.25(0.02,0.70) | -1.88(-2.19,-1.58) | 9.08(0.92,25.56) | 4.84(0.56,13.09) | -2.28(-2.74,-1.83) |
| Tonga | 1.16(0.81,1.62) | 0.85(0.58,1.23) | -1.19(-1.40,-0.97) | 0.25(0.02,0.65) | 0.17(0.02,0.45) | -1.53(-1.94,-1.12) | 5.09(0.63,12.70) | 3.39(0.48,9.30) | -1.54(-2.16,-0.90) |
| Trinidad and Tobago | 6.68(4.51,9.40) | 23.36(15.30,33.86) | 4.93(0.66,9.37) | 1.49(0.96,2.16) | 3.11(1.98,4.64) | 2.74(-1.34,6.99) | 33.77(21.91,48.40) | 70.35(45.06,104.28) | 2.73(-1.30,6.92) |
| Tunisia | 2.06(1.41,2.94) | 1.97(1.30,2.87) | 0.15(-0.45,0.75) | 0.52(0.07,1.52) | 0.48(0.06,1.47) | -0.57(-0.77,-0.36) | 9.15(1.36,24.86) | 7.20(1.19,21.03) | -0.98(-1.42,-0.53) |
| Turkmenistan | 5.97(3.59,8.74) | 4.76(2.79,7.17) | -1.15(-2.58,0.30) | 2.65(1.56,4.51) | 1.68(0.88,3.07) | -2.06(-3.76,-0.33) | 58.42(34.25,100.85) | 37.22(19.32,68.79) | -2.10(-3.77,-0.39) |
| Tuvalu | 0.73(0.50,1.02) | 0.61(0.42,0.87) | -0.68(-1.06,-0.31) | 0.45(0.04,1.20) | 0.25(0.03,0.64) | -1.95(-2.18,-1.71) | 8.81(0.90,23.07) | 4.92(0.58,12.53) | -2.03(-2.46,-1.59) |
| Turkey | 0.42(0.29,0.59) | 0.48(0.32,0.70) | 0.62(-1.96,3.27) | 0.09(0.01,0.25) | 0.06(0.01,0.15) | -1.11(-3.26,1.08) | 1.95(0.35,5.60) | 1.27(0.20,3.17) | -1.22(-3.75,1.38) |
| Uganda | 4.92(2.48,8.41) | 4.22(2.17,7.15) | 0.16(-3.60,4.06) | 0.00(0.00,0.03) | 0.00(0.00,0.02) | -1.94(-3.78,-0.06) | 0.56(0.24,1.12) | 0.44(0.20,0.87) | -0.40(-3.63,2.94) |
| Ukraine | 87.47(57.26,127.74) | 91.42(57.17,137.20) | 0.04(-0.67,0.76) | 29.99(23.69,37.25) | 29.12(20.58,39.69) | -0.47(-1.38,0.45) | 578.25(462.53,708.80) | 570.90(406.63,774.86) | -0.42(-1.48,0.67) |
| United Arab Emirates | 2.27(1.54,3.23) | 2.23(1.51,3.19) | 0.42(0.12,0.71) | 0.81(0.14,2.08) | 0.47(0.09,1.18) | 1.47(0.11,2.85) | 16.39(3.19,42.02) | 9.03(1.83,22.95) | 0.74(-0.32,1.80) |
| United Kingdom | 7.15(4.38,10.72) | 11.11(6.95,16.53) | 0.84(-0.37,2.07) | 1.87(1.72,1.99) | 1.56(1.38,1.71) | -1.93(-3.40,-0.44) | 36.42(34.10,38.58) | 33.52(30.10,36.50) | -1.48(-3.15,0.22) |
| United Republic of Tanzania | 5.22(2.60,8.98) | 4.61(2.35,7.91) | -0.04(-4.02,4.11) | 0.00(0.00,0.03) | 0.00(0.00,0.02) | -1.01(-2.85,0.85) | 0.59(0.26,1.26) | 0.50(0.22,1.08) | -0.33(-3.65,3.10) |
| United States of America | 45.88(28.91,67.34) | 50.04(33.11,71.99) | -0.33(-2.15,1.53) | 6.94(6.14,7.86) | 4.65(4.20,5.10) | -2.16(-4.26,-0.01) | 150.08(133.16,170.41) | 106.07(96.91,115.67) | -1.97(-4.04,0.15) |
| United States Virgin Islands | 18.18(12.51,25.41) | 34.00(22.53,48.80) | 2.45(0.60,4.32) | 4.10(1.45,11.11) | 3.90(0.92,8.03) | 0.40(-1.42,2.25) | 92.91(34.70,243.78) | 86.69(23.52,171.94) | 0.36(-1.43,2.18) |
| Uruguay | 54.45(33.61,81.63) | 30.09(16.85,46.12) | -3.10(-6.24,0.16) | 15.36(11.39,20.08) | 5.30(3.88,7.00) | -5.21(-8.67,-1.61) | 329.24(245.09,427.61) | 115.90(85.93,151.78) | -5.24(-8.72,-1.63) |
| Uzbekistan | 0.08(0.05,0.13) | 0.07(0.04,0.12) | -2.45(-2.98,-1.92) | 0.02(0.01,0.03) | 0.01(0.01,0.02) | -2.02(-2.79,-1.25) | 0.36(0.23,0.56) | 0.31(0.21,0.45) | -1.94(-2.67,-1.21) |
| Vanuatu | 0.68(0.47,0.95) | 0.52(0.36,0.74) | -0.94(-1.25,-0.62) | 0.36(0.04,0.90) | 0.23(0.03,0.58) | -1.71(-2.04,-1.38) | 7.48(0.94,18.84) | 4.66(0.66,11.69) | -1.84(-2.40,-1.28) |
| Venezuela (Bolivarian Republic of) | 14.62(9.82,20.80) | 5.96(3.85,8.71) | -2.15(-5.12,0.92) | 4.46(3.17,5.93) | 1.19(0.74,1.82) | -4.27(-7.53,-0.89) | 99.90(72.13,130.94) | 24.69(15.41,37.67) | -4.51(-7.86,-1.04) |
| Viet Nam | 0.93(0.63,1.32) | 1.17(0.78,1.68) | 0.29(-1.70,2.32) | 0.22(0.03,0.61) | 0.25(0.03,0.64) | 0.07(-1.32,1.47) | 4.23(0.76,11.31) | 4.63(0.71,12.05) | 0.02(-1.70,1.77) |
| Yemen | 1.46(0.99,2.11) | 1.16(0.76,1.70) | -0.48(-0.99,0.04) | 0.74(0.10,2.48) | 0.51(0.09,1.60) | -1.32(-1.53,-1.11) | 13.51(1.99,42.49) | 8.71(1.70,26.52) | -1.49(-1.87,-1.11) |
| Zambia | 5.23(2.61,9.11) | 4.28(2.17,7.36) | -0.20(-3.85,3.59) | 0.00(0.00,0.02) | 0.01(0.00,0.05) | 1.42(-0.36,3.24) | 0.57(0.25,1.12) | 0.53(0.21,1.51) | 0.12(-2.93,3.27) |
| Zimbabwe | 1.27(0.64,2.26) | 1.05(0.54,1.81) | -0.29(-3.05,2.55) | 0.22(0.03,0.87) | 0.15(0.02,0.60) | -0.68(-5.11,3.97) | 2.38(0.40,8.88) | 1.79(0.30,6.66) | -0.43(-4.76,4.10) |

**Abbreviations:** ASDR, age-standardized disability-adjusted life years rate (per 100,000 population); ASMR, age-standardized mortality rate (per 100,000 population); ASPR, age-standardized prevalence rate (per 100,000 population); CI, confidence interval; EAPC, estimated annual percentage change; UI, uncertainty interval.

**Notes:** Countries are listed alphabetically. All rates are age-standardized to the GBD 2021 global standard population. 95% uncertainty intervals (UI) are shown in parentheses for 1990 and 2021 estimates. EAPC values with 95% confidence intervals (CI) represent the average annual percentage change from 1990 to 2021. EAPC > 0 with 95% CI lower limit > 0 indicates a statistically significant increasing trend; EAPC < 0 with 95% CI upper limit < 0 indicates a statistically significant decreasing trend.

Supplementary Table 2: Decomposition Analysis Results.

|  | location | Overall_Difference | Aging | Population | Epidemiological_Change | Aging_Percentage | Population_Percentage | Epidemiological_Change_Percentage |
| --- | --- | --- | --- | --- | --- | --- | --- | --- |
| Prevalence | Southeast Asia | 495.24 | 5.49 | 455.81 | 33.94 | 1.11 | 92.04 | 6.85 |
| Prevalence | East Asia | 5813.72 | 107.08 | 3541.1 | 2165.54 | 1.84 | 60.91 | 37.25 |
| Prevalence | Global | 110487.7 | 1859.41 | 123118.5 | -14490.2 | 1.68 | 111.43 | -13.11 |
| Prevalence | Oceania | 2.9 | 0.17 | 3.31 | -0.57 | 5.73 | 114.06 | -19.78 |
| Prevalence | Central Asia | 552.06 | 8.53 | 365.04 | 178.49 | 1.55 | 66.12 | 32.33 |
| Prevalence | Eastern Europe | 29355.39 | 671.2 | 12887.83 | 15796.36 | 2.29 | 43.9 | 53.81 |
| Prevalence | Central Europe | 12111.71 | 401.46 | 5871.14 | 5839.1 | 3.31 | 48.47 | 48.21 |
| Prevalence | Western Europe | 19636.24 | 21.14 | 15893.23 | 3721.88 | 0.11 | 80.94 | 18.95 |
| Prevalence | Australasia | 5617.13 | -27.47 | 2716.58 | 2928.02 | -0.49 | 48.36 | 52.13 |
| Prevalence | High-income Asia Pacific | 2182.85 | -293.35 | 2440.84 | 35.37 | -13.44 | 111.82 | 1.62 |
| Prevalence | High-income North America | 25269.81 | -81.74 | 20598.26 | 4753.29 | -0.32 | 81.51 | 18.81 |
| Prevalence | Caribbean | 3594.06 | -23.54 | 1247.49 | 2370.11 | -0.65 | 34.71 | 65.95 |
| Prevalence | Southern Latin America | -308.31 | -2.23 | 677.41 | -983.5 | 0.72 | -219.72 | 318.99 |
| Prevalence | Andean Latin America | 16.71 | 0.53 | 15.13 | 1.05 | 3.14 | 90.57 | 6.29 |
| Prevalence | Tropical Latin America | 1656.45 | -36.54 | 3921.28 | -2228.28 | -2.21 | 236.73 | -134.52 |
| Prevalence | Central Latin America | 1120.58 | 5.15 | 1112.91 | 2.51 | 0.46 | 99.32 | 0.22 |
| Prevalence | North Africa and Middle East | 261.32 | 3.1 | 284.11 | -25.88 | 1.19 | 108.72 | -9.91 |
| Prevalence | South Asia | 1952.21 | 75.97 | 1861.62 | 14.62 | 3.89 | 95.36 | 0.75 |
| Prevalence | Central Sub-Saharan Africa | 101.19 | -0.53 | 120.45 | -18.73 | -0.53 | 119.04 | -18.52 |
| Prevalence | Eastern Sub-Saharan Africa | 439.48 | -6.45 | 495.83 | -49.9 | -1.47 | 112.82 | -11.35 |
| Prevalence | Western Sub-Saharan Africa | 610.13 | -6.76 | 567.4 | 49.49 | -1.11 | 93 | 8.11 |
| Prevalence | Southern Sub-Saharan Africa | 6.85 | -1.91 | 15.38 | -6.62 | -27.92 | 224.61 | -96.7 |
| Prevalence | Middle SDI | 9259.12 | 154.38 | 7361.4 | 1743.34 | 1.67 | 79.5 | 18.83 |
| Prevalence | Low SDI | 1199.91 | 2.39 | 1168.45 | 29.07 | 0.2 | 97.38 | 2.42 |
| Prevalence | Low-middle SDI | 2524.73 | 56.05 | 3056.63 | -587.94 | 2.22 | 121.07 | -23.29 |
| Prevalence | High SDI | 59761.14 | -293.25 | 51514.79 | 8539.61 | -0.49 | 86.2 | 14.29 |
| Prevalence | High-middle SDI | 37436.45 | 390.39 | 41342.58 | -4296.53 | 1.04 | 110.43 | -11.48 |
| Deaths | High-income Asia Pacific | 28.46 | 18.94 | 293.53 | -284 | 66.52 | 1031.2 | -997.72 |
| Deaths | Central Asia | 155.15 | -1.87 | 118.04 | 38.98 | -1.21 | 76.09 | 25.12 |
| Deaths | High-income North America | 792.48 | -26.31 | 2356.57 | -1537.78 | -3.32 | 297.37 | -194.05 |
| Deaths | Global | 6373.33 | 1071.57 | 22001.08 | -16699.3 | 16.81 | 345.21 | -262.02 |
| Deaths | Central Sub-Saharan Africa | 0.29 | 0 | 0.43 | -0.13 | -0.75 | 145.36 | -44.61 |
| Deaths | Oceania | 0.67 | 0.05 | 1.17 | -0.56 | 7.91 | 175.03 | -82.95 |
| Deaths | High SDI | -661.31 | 358.22 | 6840.39 | -7859.91 | -54.17 | -1034.37 | 1188.54 |
| Deaths | High-middle SDI | 5656.31 | 514.82 | 9819.34 | -4677.85 | 9.1 | 173.6 | -82.7 |
| Deaths | Andean Latin America | 0.68 | 0.11 | 2.94 | -2.37 | 16.17 | 429.52 | -345.69 |
| Deaths | Western Sub-Saharan Africa | -2.89 | 0.51 | 28.06 | -31.46 | -17.81 | -971.72 | 1089.53 |
| Deaths | Tropical Latin America | -178.17 | -9.94 | 754.04 | -922.27 | 5.58 | -423.22 | 517.64 |
| Deaths | Low-middle SDI | 390.47 | 14.47 | 620.13 | -244.13 | 3.7 | 158.82 | -62.52 |
| Deaths | East Asia | 624.4 | 56.35 | 429.81 | 138.25 | 9.02 | 68.84 | 22.14 |
| Deaths | Australasia | 115.2 | 13.19 | 195.19 | -93.19 | 11.45 | 169.44 | -80.9 |
| Deaths | Eastern Europe | 5148.18 | 264.74 | 2967.89 | 1915.56 | 5.14 | 57.65 | 37.21 |
| Deaths | Caribbean | 482.33 | 0.47 | 181.25 | 300.61 | 0.1 | 37.58 | 62.32 |
| Deaths | Low SDI | 92.09 | 2.83 | 107.37 | -18.11 | 3.08 | 116.59 | -19.67 |
| Deaths | North Africa and Middle East | 41 | 3.17 | 73.72 | -35.9 | 7.74 | 179.83 | -87.57 |
| Deaths | Western Europe | -2185.44 | 429.52 | 2324.24 | -4939.21 | -19.65 | -106.35 | 226.01 |
| Deaths | Southeast Asia | 78.72 | 2.78 | 95.72 | -19.78 | 3.54 | 121.59 | -25.13 |
| Deaths | Middle SDI | 870.13 | 50.87 | 1277.93 | -458.67 | 5.85 | 146.87 | -52.71 |
| Deaths | Southern Sub-Saharan Africa | 0.27 | -0.38 | 1.14 | -0.49 | -143.25 | 428.96 | -185.71 |
| Deaths | Central Latin America | 102.48 | 2.33 | 169.98 | -69.83 | 2.28 | 165.87 | -68.14 |
| Deaths | Eastern Sub-Saharan Africa | 0.22 | 0 | 0.37 | -0.15 | -0.43 | 168.89 | -68.46 |
| Deaths | Central Europe | 985.65 | 248.41 | 1297.32 | -560.08 | 25.2 | 131.62 | -56.82 |
| Deaths | South Asia | 399.5 | 14.99 | 450.94 | -66.42 | 3.75 | 112.88 | -16.63 |
| Deaths | Southern Latin America | -215.86 | 1.7 | 175.77 | -393.33 | -0.79 | -81.43 | 182.22 |
| DALYs | Low SDI | 2031.89 | -10.05 | 2456.91 | -414.96 | -0.49 | 120.92 | -20.42 |
| DALYs | Global | 180752.9 | -6052.93 | 467845.2 | -281039 | -3.35 | 258.83 | -155.48 |
| DALYs | Tropical Latin America | -4177.76 | -615.03 | 17851.26 | -21414 | 14.72 | -427.29 | 512.57 |
| DALYs | South Asia | 8211.77 | 22.51 | 9913.82 | -1724.56 | 0.27 | 120.73 | -21 |
| DALYs | Middle SDI | 17529.73 | -338.59 | 28477.36 | -10609 | -1.93 | 162.45 | -60.52 |
| DALYs | East Asia | 12369.38 | 173.67 | 9122.38 | 3073.33 | 1.4 | 73.75 | 24.85 |
| DALYs | High-income Asia Pacific | -213.64 | -613.36 | 6203.03 | -5803.31 | 287.1 | -2903.55 | 2716.44 |
| DALYs | Central Europe | 25915.53 | -10.91 | 25683.02 | 243.42 | -0.04 | 99.1 | 0.94 |
| DALYs | High-income North America | 22099.64 | -33.46 | 51367.69 | -29234.6 | -0.15 | 232.44 | -132.29 |
| DALYs | Central Latin America | 2216.74 | -30.98 | 3793.16 | -1545.43 | -1.4 | 171.11 | -69.72 |
| DALYs | High-middle SDI | 149250.5 | -10570.4 | 215673.8 | -55852.9 | -7.08 | 144.5 | -37.42 |
| DALYs | Eastern Europe | 136286.6 | -7805.7 | 67859.61 | 76232.65 | -5.73 | 49.79 | 55.94 |
| DALYs | Caribbean | 10323.46 | -98.78 | 3982.37 | 6439.87 | -0.96 | 38.58 | 62.38 |
| DALYs | Central Asia | 3863.4 | 97.09 | 2652.78 | 1113.52 | 2.51 | 68.66 | 28.82 |
| DALYs | High SDI | 3051.08 | -2893.08 | 138731.1 | -132787 | -94.82 | 4546.95 | -4352.13 |
| DALYs | North Africa and Middle East | 730.81 | 14.26 | 1515.49 | -798.94 | 1.95 | 207.37 | -109.32 |
| DALYs | Eastern Sub-Saharan Africa | 45.79 | -0.7 | 54.34 | -7.85 | -1.54 | 118.68 | -17.15 |
| DALYs | Western Sub-Saharan Africa | 2.89 | -8.83 | 598.49 | -586.78 | -305.05 | 20687.33 | -20282.3 |
| DALYs | Low-middle SDI | 8253.7 | -113.79 | 13885.88 | -5518.39 | -1.38 | 168.24 | -66.86 |
| DALYs | Southeast Asia | 1697.73 | 15.7 | 2124.74 | -442.71 | 0.92 | 125.15 | -26.08 |
| DALYs | Australasia | 2647.22 | -44.63 | 4082.26 | -1390.4 | -1.69 | 154.21 | -52.52 |
| DALYs | Oceania | 15.36 | 0.37 | 28.06 | -13.07 | 2.41 | 182.69 | -85.1 |
| DALYs | Andean Latin America | 11.81 | 1.75 | 50.04 | -39.99 | 14.86 | 423.78 | -338.64 |
| DALYs | Southern Sub-Saharan Africa | 4.84 | -3.49 | 13.9 | -5.57 | -72.1 | 287.3 | -115.19 |
| DALYs | Western Europe | -36348.8 | 128.9 | 44226.85 | -80704.6 | -0.35 | -121.67 | 222.03 |
| DALYs | Southern Latin America | -4965.72 | -160.61 | 3855.54 | -8660.65 | 3.23 | -77.64 | 174.41 |
| DALYs | Central Sub-Saharan Africa | 15.89 | -0.24 | 20.57 | -4.44 | -1.53 | 129.45 | -27.93 |

Supplementary Table 3: Predictive Analysis Results.

| Year | ASPR | | | ASMR | | | ASDR | | |
| --- | --- | --- | --- | --- | --- | --- | --- | --- | --- |
|  | val | 95%CI_lower | 95%CI_upper | val | 95%CI_lower | 95%CI_upper | val | 95%CI_lower | 95%CI_upper |
| 2022 | 20.027218 | 18.628161 | 21.426274 | 2.667954 | 2.3226516 | 3.0132564 | 58.847394 | 50.481809 | 67.212979 |
| 2023 | 20.121519 | 18.370923 | 21.872115 | 2.6518669 | 2.2022345 | 3.1014994 | 58.576038 | 47.905995 | 69.24608 |
| 2024 | 20.21274 | 18.107207 | 22.318274 | 2.6395104 | 2.0923993 | 3.1866215 | 58.370426 | 45.41106 | 71.329792 |
| 2025 | 20.298383 | 17.808546 | 22.78822 | 2.6300864 | 1.9844534 | 3.2757194 | 58.209216 | 42.840519 | 73.577914 |
| 2026 | 20.385754 | 17.45789 | 23.313619 | 2.6199429 | 1.8705581 | 3.3693277 | 58.04471 | 40.064352 | 76.025069 |
| 2027 | 20.492666 | 17.051489 | 23.933843 | 2.610827 | 1.748738 | 3.4729159 | 57.893052 | 37.037026 | 78.749078 |
| 2028 | 20.60532 | 16.573507 | 24.637134 | 2.6029842 | 1.6182008 | 3.5877675 | 57.763665 | 33.757942 | 81.769388 |
| 2029 | 20.717701 | 16.012655 | 25.422746 | 2.5972401 | 1.4783843 | 3.7160958 | 57.661587 | 30.220326 | 85.102847 |
| 2030 | 20.827591 | 15.35927 | 26.295912 | 2.5930207 | 1.3277665 | 3.858275 | 57.580128 | 26.407319 | 88.752938 |
| 2031 | 20.94167 | 14.610765 | 27.272575 | 2.588863 | 1.1640931 | 4.013633 | 57.499929 | 22.292384 | 92.707474 |
| 2032 | 21.069717 | 13.77 | 28.369434 | 2.5853439 | 0.9871453 | 4.1835424 | 57.429703 | 17.877319 | 96.982087 |
| 2033 | 21.205261 | 12.834909 | 29.575613 | 2.5831719 | 0.7975671 | 4.3687767 | 57.37525 | 13.178165 | 101.57233 |
| 2034 | 21.342193 | 11.8016 | 30.882787 | 2.5821908 | 0.595304 | 4.5690775 | 57.337061 | 8.2018297 | 106.47229 |
| 2035 | 21.478722 | 10.667414 | 32.29003 | 2.5825539 | 0.3799985 | 4.7851094 | 57.311958 | 2.9473945 | 111.67652 |
| 2036 | 21.614893 | 9.4307788 | 33.799007 | 2.5827741 | 0.1503037 | 5.0152445 | 57.287756 | -2.594172 | 117.16968 |
| 2037 | 21.756618 | 8.0952744 | 35.417961 | 2.5837599 | -0.093559 | 5.2610791 | 57.26971 | -8.416776 | 122.9562 |
| 2038 | 21.900596 | 6.6612818 | 37.139911 | 2.5855045 | -0.350851 | 5.5218595 | 57.260121 | -14.50582 | 129.02606 |
| 2039 | 22.046107 | 5.1289958 | 38.963218 | 2.5883596 | -0.621345 | 5.7980642 | 57.259743 | -20.85272 | 135.37221 |
| 2040 | 22.191432 | 3.4975513 | 40.885312 | 2.5917152 | -0.905104 | 6.0885348 | 57.265193 | -27.45331 | 141.9837 |
| 2041 | 22.33847 | 1.7673095 | 42.909631 | 2.5952973 | -1.202759 | 6.3933536 | 57.271907 | -34.30771 | 148.85152 |
| 2042 | 22.48864 | -0.060279 | 45.03756 | 2.5990105 | -1.513943 | 6.7119638 | 57.281003 | -41.40914 | 155.97114 |
| 2043 | 22.642045 | -1.984671 | 47.268761 | 2.6034818 | -1.838244 | 7.0452074 | 57.295221 | -48.74808 | 163.33852 |
| 2044 | 22.796715 | -4.006322 | 49.599753 | 2.6082859 | -2.175248 | 7.3918196 | 57.313274 | -56.31775 | 170.9443 |
| 2045 | 22.952798 | -6.12646 | 52.032055 | 2.6135067 | -2.525338 | 7.7523513 | 57.333753 | -64.1155 | 178.783 |
| 2046 | 23.109477 | -8.345515 | 54.56447 | 2.6184356 | -2.888446 | 8.1253173 | 57.353123 | -72.13862 | 186.84486 |
| 2047 | 23.268609 | -10.66362 | 57.200836 | 2.6234922 | -3.26483 | 8.5118139 | 57.372562 | -80.38344 | 195.12857 |
| 2048 | 23.428986 | -13.07983 | 59.937801 | 2.6284532 | -3.653676 | 8.9105823 | 57.392332 | -88.84227 | 203.62693 |
| 2049 | 23.591282 | -15.59463 | 62.777194 | 2.6337201 | -4.055218 | 9.3226583 | 57.413567 | -97.51097 | 212.33811 |
| 2050 | 23.753973 | -18.20753 | 65.715477 | 2.6388184 | -4.468683 | 9.7463195 | 57.435068 | -106.3841 | 221.25421 |

**Abbreviations:** ASDR, age-standardized disability-adjusted life years rate (per 100,000 population); ASMR, age-standardized mortality rate (per 100,000 population); ASPR, age-standardized prevalence rate (per 100,000 population); CI, confidence interval; DALYs, disability-adjusted life years.

**Notes:** Projections for 2022–2050 were generated using a Bayesian age-period-cohort (BAPC) model with integrated nested Laplace approximation (INLA). All rates are age-standardized to the GBD 2021 global standard population. 95% confidence intervals (CI) represent the uncertainty range of the projections. Projections assume continuation of current trends and do not account for potential future policy changes or public health interventions.
